# A damaged genome’s transcriptional landscape through multilayered expression profiling around *in situ*-mapped DNA double-strand breaks

**DOI:** 10.1038/ncomms15656

**Published:** 2017-05-31

**Authors:** Fabio Iannelli, Alessandro Galbiati, Ilaria Capozzo, Quan Nguyen, Brian Magnuson, Flavia Michelini, Giuseppina D’Alessandro, Matteo Cabrini, Marco Roncador, Sofia Francia, Nicola Crosetto, Mats Ljungman, Piero Carninci, Fabrizio d’Adda di Fagagna

**Affiliations:** 1 IFOM, The FIRC Institute of Molecular Oncology, Via Adamello 16, 20139 Milan, Italy; 2 IGM (Istituto di Genetica Molecolare)-CNR (Consiglio Nazionale delle Ricerche). Via Abbiategrasso 207, 27100 Pavia, Italy; 3 Division of Genomic Technologies, RIKEN Center for Life Science Technologies, 1-7-22 Suehiro-cho, Tsurumi-ku, Yokohama, 230-0045, Japan; 4 Department of Radiation Oncology, University of Michigan, Ann Arbor, Michigan 48109, USA; 5 Science for Life Laboratory, Division of Translational Medicine and Chemical Biology, Department of Medical Biochemistry and Biophysics, Karolinska Institutet, 171 65 Stockholm, Sweden

## Abstract

Of the many types of DNA damage, DNA double-strand breaks (DSBs) are probably the most deleterious. Mounting evidence points to an intricate relationship between DSBs and transcription. A cell system in which the impact on transcription can be investigated at precisely mapped genomic DSBs is essential to study this relationship. Here in a human cell line, we map genome-wide and at high resolution the DSBs induced by a restriction enzyme, and we characterize their impact on gene expression by four independent approaches by monitoring steady-state RNA levels, rates of RNA synthesis, transcription initiation and RNA polymerase II elongation. We consistently observe transcriptional repression in proximity to DSBs. Downregulation of transcription depends on ATM kinase activity and on the distance from the DSB. Our study couples for the first time, to the best of our knowledge, high-resolution mapping of DSBs with multilayered transcriptomics to dissect the events shaping gene expression after DSB induction at multiple endogenous sites.

## Abstract (web summary)

DNA double strand breaks (DSBs) are among the most deleterious types of damage and there is strong evidence indicating a relationship between breaks and transcription. Here the authors provide a high-resolution, genome-wide map of induced DSBs and observe ATM-dependent transcriptional repression.

The DNA damage response (DDR) is a complex signalling cascade that coordinates cellular DNA-repair activities following DNA-damage detection, while transiently arresting cell cycle progression until lesions have been fully removed^1^. DNA damage in the form of a DNA double-strand break (DSB) is sensed by specialized sensor complexes that recruit and activate at the site of damage three members of the phosphatidylinositol 3-kinase-related kinase family: ataxia-telangiectasia mutated (ATM), ataxia-telangiectasia and Rad3-related and DNA-dependent protein kinase (DNA-PK). These proteins are responsible for the phosphorylation at Ser139 of the histone variant H2AX, known as *γ*H2AX, a key step in the DDR cascade, with ATM being the primary kinase responsible for this modification upon DSB and working redundantly with DNA-PK.

The DDR ultimately alters gene expression via multiple transcriptional pathways that are engaged to promote cell cycle arrest and DNA repair events^2^. Interestingly, activation of the DDR is emerging as a key factor also regulating transcriptional events at the site of DNA damage^3^. DDR activation has been recently shown to be involved in the suppression of transcription of damaged transcriptional units or adjacent to DSBs in mammalian cells^4^,^5^,^6^. Although this phenomenon deserves further investigation, important links have already been discovered between transcriptional silencing and the DDR kinases ATM and DNA-PK. Notably, inhibition of ATM or DNA-PK has been reported to restore pre-existing transcription in the presence of a DSB^4^,^5^, suggesting that the observed repressive response is not due to the lesion *per se*, but it is actively regulated by the DDR pathways.

A cluster of DSBs induced by the FokI endonuclease has been reported to repress transcription of a distal artificial reporter gene in *cis* in an ATM-dependent manner^5^. In contrast, the generation of individual DNA breaks within specific genes (*DAB1* and *RYR2*) by I-PpoI endonuclease does not seem to affect the transcription of neighbouring transcriptional units, whereas inhibiting transcription of the specific DSB-carrying gene in a DNA-PK-dependent manner^4^. The observation that DSBs within, but not adjacent to, transcriptional units may inhibit transcription is consistent with seemingly unaltered presence of RNA polymerase II (RNAPII) within DSB-induced γH2AX-containing chromatin regions^7^. Finally, a recent study based on RNA sequencing (RNA-seq) in a mouse model that allows controlled DSB formation by I-PpoI endonuclease, an enzyme that induces DSBs mainly in ribosomal genes, thus in a repetitive cluster, showed transient, and ATM- and DNA-PK-dependent transcriptional repression of genes proximal to the breaks^6^.

However, the impact of DSBs on the different mechanisms of transcription regulation, such as the rates of RNA synthesis and transcription initiation events, has, to our knowledge, never been reported.

The impact of DSB on transcription is further compounded by recent reports demonstrating the induction, rather than the suppression, of transcription. Indeed, short non-coding RNAs have been reported to be generated at DSBs and involved in DDR activation and DNA repair, and were thus named DDR RNAs (DDRNAs)^8^ and DSB-induced RNAs^9^. The detection of DDRNAs with the sequence of the DNA adjacent to the DSB site, and the participation of DICER and DROSHA endoribonucleases to their biogenesis, strongly suggests the presence of transcription near the DSB to allow the synthesis of a longer precursor RNA^10^.

Thus, the relationship between DNA damage and transcription appears to be more complicated than expected and an approach inspecting physiological DSBs distributed genome-wide would be desirable.

Here we assessed the transcriptional landscape near sites of DNA damage by high-resolution genome-wide mapping of DSBs coupled with transcriptome profiling in the human cell line DIvA (*Asi*SI-ER-U20S^7^), which stably expresses the fusion *Asi*SI-ER restriction enzyme. This system allows the inducible generation of DSBs at only a subset of predicted *Asi*SI recognition sites in the genome, thus providing a convenient internal reference made of uncut sites bearing the same sequence. Differently from other cell systems previously used^4^,^6^, *Asi*SI recognition sites are broadly distributed among non-repetitive sequences of the genome.

Studying the transcriptional landscape around DSBs requires their precise genomic mapping. The most commonly used method to study DSB location, chromatin immunoprecipitation followed by genome-wide sequencing (ChIP-seq), has relevant drawbacks. It relies on the detection of DDR factors accumulating near DSBs as a proxy for DNA damage and it is known that DDR factors recruitment to DNA lesions can be impaired by the chromatin structure^11^,^12^,^13^. Moreover, phosphorylation of H2AX can spread up to 1 Mb away from a DSB, making the precise mapping of the DSB unattainable. The recent observation that multiple DSBs may associate within repair centres^14^ could affect the correct determination of the number of DDR foci and possibly also the mapping of DSB by ChIP-seq approaches based on DDR markers.

To accurately identify the DSBs induced by *Asi*SI, we applied our recently developed method for breaks labelling *in situ* and sequencing (BLISS)^15^. BLISS greatly expands the quantitative nature, sensitivity and versatility of the original direct *in situ* breaks labelling, enrichment on streptavidin, and sequencing (BLESS) method for genome-wide direct DSB detection^16^ and its recent modifications^17^,^18^, by allowing direct labelling and amplification of DSB ends *in situ*. In addition, to independently assess *Asi*SI cut sites with an established and routinely used technology, we performed *γ*H2AX ChIP-seq experiments. By matching BLISS-detected *Asi*SI cut sites with those identified by ChIP-seq, we obtained a set of high-confidence DSBs confirmed by both technologies. Next, we took advantage of this precise genome-wide map of DSBs to study the impact of DNA damage on pre-existing transcription by profiling the transcriptional landscape of *Asi*SI-induced and uninduced cells. Transcription alterations after DNA damage induction may be determined by various biological mechanisms, including changes in transcription initiation, RNA synthesis, RNA elongation or all three together. To distinguish among these potential regulatory steps, we used a set of four independent genome-wide sequencing technologies: RNA-seq, Bru-seq (metabolic pulse-labelling of RNA with bromouridine (Bru) followed by sequencing), CAGE (cap-analysis of gene expression) and ChIP-seq of elongating RNAPII. RNA-seq allows the identification of differentially expressed genes, Bru-seq quantifies differential rates of RNA synthesis, CAGE maps transcription start activities and ChIP-seq shows the different levels of total and elongating RNAPII following DSB induction. The results obtained were independently validated by reverse-transcription quantitative PCR (RT–qPCR) at individual cut sites.

## Results

### Identification of *Asi*SI cut sites

We used DIvA, a human osteosarcoma cell line (U2OS) stably expressing the *Asi*SI-ER fusion protein where the AsiSI restriction enzyme is fused to a modified oestrogen receptor (ER) hormone-binding domain, which only binds to 4-hydroxy tamoxifen (4OHT)^7^. The addition of 4OHT to the cell culture growth medium induces the nuclear localization of *Asi*SI-ER, generating many sequence-specific DSBs at predictable genomic loci (Supplementary Fig. 1a–c).

To compute a map of *Asi*SI restriction sites present in the human genome, we performed an *in silico* digestion of the GRCh37/hg19 human genome assembly. We detected a total of 1,242 restriction sites: 1,219 mapped on reference chromosomes (0.5 cut sites per Mb on average, Supplementary Fig. 2a), whereas the remaining ones mapped on haplotype chromosomes and unplaced contigs of the hg19 assembly.

To identify the *Asi*SI target sequences that were most efficiently cut upon translocation of the enzyme in the nucleus, we performed BLISS on *Asi*SI-induced and uninduced cells (Supplementary Fig. 1a). We focused on windows of ±100 bp surrounding an *Asi*SI recognition site and applied a normalization procedure of the number of reads surrounding these windows in induced and uninduced samples, to rank the cutting efficiency at individual *Asi*SI sites (see Methods). We detected 214 *Asi*SI sites ranked on the basis of read enrichment in induced cells with respect to uninduced cells (Supplementary Table 1). Among these, we identified as efficiently cut 113 *Asi*SI never reported before in previous publications employing this cell system^18^.

**Figure 1:**
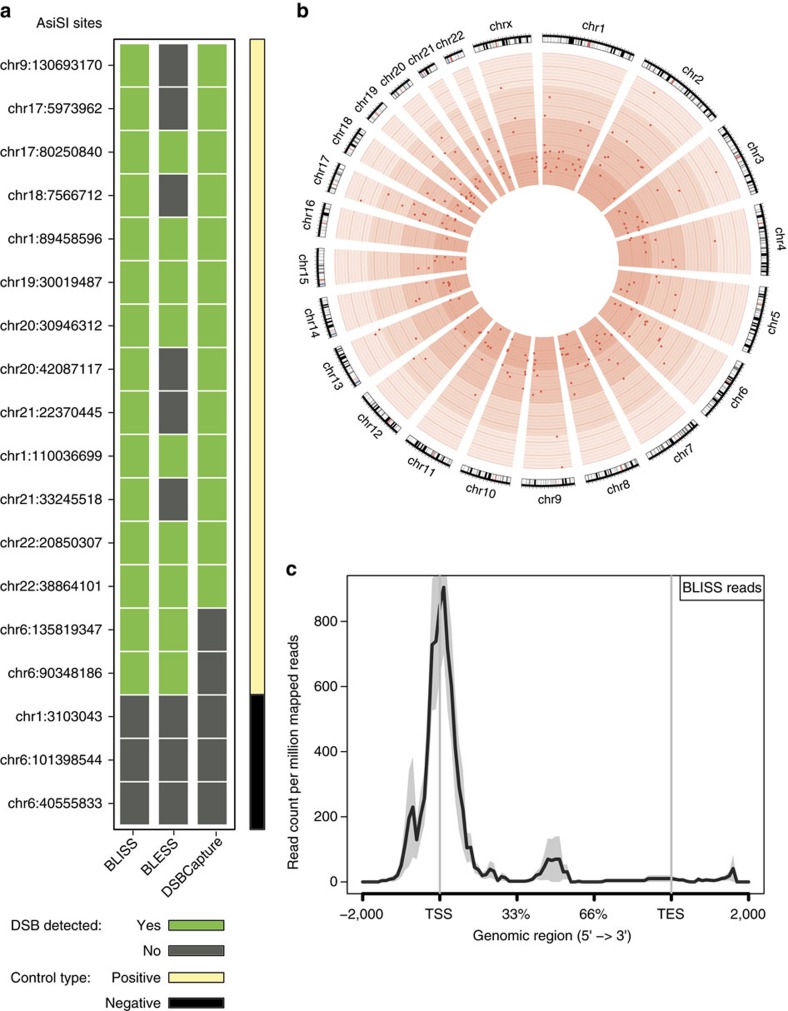
Characterization of *Asi*SI sites detected by BLISS. (**a**) BLISS^15^, BLESS^16^ and DSBCapture^18^—a DSB detection method based on BLESS—efficiency in detecting cut *Asi*SI sites calculated for a subset of *Asi*SI sites reported in the literature (see Supplementary Table 2 for references) to be cut upon 4OHT treatment of DIvA cells. (**b**) The circos plot (http://circos.ca/) shows for each chromosome the *Asi*SI sites detected by BLISS. Each dot represents an *Asi*SI site, whereas circles with different shades of red denote the read coverage density of BLISS reads in a±100 bp window of each of AsiSI site (min density=0, max density=0.85; bins of read density from inner to outer circle: 0–0.25, 0.25–0.50, 0.50–0.75, 0.75–1.00). (**c**) Coverage profile plot representing the read count per million mapped reads (RPM) of *Asi*SI sites detected by BLISS for each genebody region. TES, transcription end site; TSS, transcription start site. Bold lines represent mean value, whereas the semi-transparent shades around the mean curve represent the s.e.m. across the regions.

To independently validate our BLISS data, we performed ChIP-seq for γH2AX in induced and uninduced DIvA cells to evaluate the efficiency of DSB induction and DDR activation at each *Asi*SI site. As expected, γH2AX showed a typical pattern^7^, with wide spreading away from the DSBs (∼1–2 Mb) and a signal dip near (∼1 Kb) the expected cut sites, presumably reflecting reduced nucleosome occupancy. We determined the subset of the 100 most efficiently cleaved *Asi*SI sites in the genome on the basis of γH2AX enrichment around the DSB (Supplementary Fig. 2b). We then performed an intersection of the 214 *Asi*SI sites detected by BLISS with those ranked among the top 100 cut sites detected by γH2AX ChIP-seq experiments. Out of the 214 *Asi*SI sites identified by BLISS, 74 were also present in γH2AX ChIP-seq data among the 100 most cleaved (Supplementary Table 1 and Supplementary Fig. 3a). Additional analyses of the 26 sites detected only by γH2AX ChIP-seq suggested they might be poorly cut and thus borderline affected in different experiments (see Supplementary Information for details and Supplementary Fig. 3). To further assess the sensitivity and specificity of BLISS in detecting *Asi*SI cut sites, we compared our results with those previously reported for the 18 *Asi*SI sites detected in DIvA cells by ligation-mediated qPCR: 15 *Asi*SI-induced DSBs (positive controls) and 3 uncut *Asi*SI sites (negative controls)^7^,^19^,^20^. In addition, we performed an independent experiment using the previously published method BLESS^16^. As shown in Fig. 1a and Supplementary Table 2, BLISS was able to detect all the 15 positive control sites tested and none of the negative control ones, demonstrating the high sensitivity and specificity of this method, superior to the BLESS method and to other approaches based on BLESS^18^ (Fig. 1a).

Next, we characterized the entire set of the 214 cut sites detected by BLISS. First, we analysed the genome-wide distribution of the cut *Asi*SI sites. As shown in Fig. 1b, these sites were equally distributed along the genome and the coverage of BLISS reads was almost uniform, underlying the absence of regions of the genome under- or over-represented because of sequence-dependent coverage variations or sequencing errors. Second, we investigated the abundance of *Asi*SI sites across gene bodies, that is, from transcription start sites (TSSs) to transcription end sites—see Methods for details. To do this, we computed BLISS read density profiles of the 214 detected cut sites across gene bodies and observed that reads were enriched at TSSs, showing that the majority of detected cut *Asi*SI sites are located in proximity to TSS (Fig. 1c). This same feature was observed by plotting all predicted 1,219 *Asi*SI sites (Supplementary Fig. 4a), demonstrating that this is not a bias of the BLISS technique—the preference for *Asi*SI to cut close to TSS is likely to be due to the GC-rich sequence recognised by the enzyme (5′-GCGATCGC-3′), which is more abundant in proximity to TSS because of the higher number of CpG dinucleotides in mammalian promoter regions^21^. Moreover, detected cut sites are significantly enriched next to genes (178 out of 214 *Asi*SI sites, *P*<4e^−10^ binomial test, Supplementary Table 1), where chromatin tends to be more open and accessible to enzymatic activities. Indeed, such sites tend to lay in proximity to DNase I hypersensitive sites, regions of chromatin sensitive to cleavage by the DNase I enzyme that are characterized by open, accessible chromatin (Supplementary Fig. 4b). Thus, the fact that not all genomic sequences containing the *Asi*SI recognition sequence are detected as cut by BLISS is not surprising and it is consistent with ChIP followed by microarray hybridization and ChIP-seq results previously reported^7^,^19^.

We thus provide, to the best of our knowledge, the most extensive and specific list of DSBs induced by a restriction enzyme (AsiSI) in a human cell line reported up to now, a resource available for additional studies among interested scientists.

### DSB induction causes inhibition of nearby transcription

**Figure 2:**
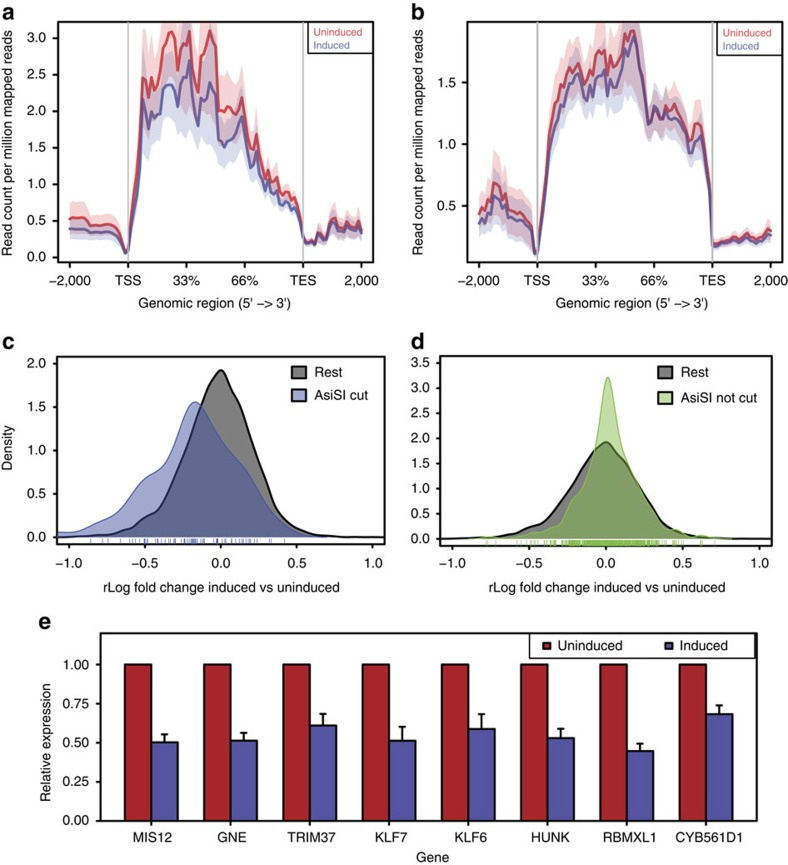
Induced cut sites lead to downregulation of pre-existing transcription. (**a**) Coverage profile plot representing the RPM of transcripts with *Asi*SI sites cut according to BLISS in induced (blue) and uninduced (red) samples for each genebody region: TES, transcription end site; TSS, transcription start site. Bold lines represent mean values, whereas semi-transparent shades around the mean curves represent the s.e.m. across the regions. (**b**) Coverage profile plot representing the RPM of transcripts with *Asi*SI sites uncut according to BLISS in induced (blue) and uninduced (red) samples for each genebody region. TES, transcription end site; TSS, transcription start site. (**c**) Distribution of the regularized-logarithm (rlog) fold change for genes with *Asi*SI sites cut according to BLISS and γH2AX ChIP-seq (blue) compared with the one of the rest of the genes (grey). Genes next to active *Asi*SI cut sites show significant shift towards downregulation (*P*<0.05, Wilcoxon test). (**d**) Distribution of the rlog fold change for genes with *Asi*SI sites uncut according to BLISS and γH2AX ChIP-seq (green) compared with the one of the rest of the genes (grey). Genes next to uncut *Asi*SI sites do not show significant shift towards downregulation (*P*=0.9996, Wilcoxon test). (**e**) qPCR validation of relative expression of eight randomly selected genes in induced (blue) versus uninduced (red) samples. Shown is the mean of biological replicates (*n*=3). Error bars show s.e.m. All reported differences where statistically significant (*P*<0.05; unpaired t test with Welch’s correction).

The precise mapping of *Asi*SI DSBs allowed us to study the effect of DSBs on the expression of genes near the induced DSBs. To this aim, we performed RNA-seq on uninduced and induced DIvA cells. We checked for evidence of alteration of RNA expression in proximity to all BLISS*-*detected *Asi*SI sites that were within or adjacent (±2 Kb) to gene bodies (178 out of 214 *Asi*SI sites, 196 genes; Supplementary Table 1). Coverage profile plots of the gene body provided evidence of downregulation of steady-state RNA as indicated by an overall reduction of reads per million mapped (RPM) values in induced cells with respect to uninduced ones (Fig. 2a). As a control, we analysed a data set of 597 genes overlapping or located in proximity (±2 Kb) to uncut *Asi*SI sites and observed no substantial difference between induced and uninduced cells, providing evidence that only cut sites are responsible for transcriptional repression (Fig. 2b).

To test for statistical significance and robustness of our observations, we profiled the gene expression of what we defined a gold standard data set of 75 genes overlapping or located in proximity (±2 Kb) to the *Asi*SI sites detected by both BLISS and γH2AX ChIP-seq (Supplementary Table 1), and of the rest of the human genes.

First, we compared differential expression (DE) values between induced and uninduced samples among defined categories of genes in bulk as cumulative distribution: those genes hit by or adjacent to (±2 Kb) one of the gold standard set of *Asi*SI sites and all other genes devoid of *Asi*SI site. As depicted in Fig. 2c, the distribution of the fold change of expression of cut genes is significantly shifted towards lower values with respect to the rest of the genes (*P*<0.05, Wilcoxon test), showing that induced DNA damage leads to downregulation of steady-state RNA levels. Interestingly, out of the genes overlapping or located in proximity to cut sites, the ones that showed no transcriptional alteration had lower BLISS read coverage per *Asi*SI site (Supplementary Fig. 4c), thus suggesting that these analyses may underestimate the downregulation effect of DSBs due to the lower cutting efficiency of the enzyme at particular genomic loci.

As an additional control, we compared the cumulative distribution of DE values of genes overlapping or located in proximity (±2 Kb) to uncut *Asi*SI sites with the distribution of the rest of human genes and we did not observe any significant downregulation, further confirming that only the cut sites are associated with repression of gene expression (Fig. 2d).

Finally, to independently validate with a different technique the results obtained so far, we designed primers in the gene body of 8 randomly selected genes and performed RT–qPCR experiments in three biological and three technical replicates. We observed a statistically significant downregulation of gene expression in all tested genes, thus independently validating the conclusions based on our computational analyses (Fig. 2e, *P*<0.05 Welch’s *t*-test).

### Transcription inhibition is independent from DSB location

The advantage of the system we used to generate DNA damage is that induced DSBs are evenly distributed throughout the genome (Fig. 1b). However, AsiSI sites are enriched next to TSS (Fig. 1c), increasing the possibility that a DSB could impair regulatory elements near the TSS. We thus investigated whether the transcription suppression observed in this system is only consequent to the potential disruption of promoter integrity and/or transcription factors binding, or if it is observed independently from the location of the DSB with respect to the transcriptional unit. To do this, we analysed two distinct subsets of DSBs: those located in the promoter region or in proximity to the TSS (that is, up to 2 Kbp upstream the TSS or in the 5′-untranslated region (UTR) exons) and those located in the rest of the transcribed region (that is, introns, coding exons or 3′-UTR exons). In both subgroups, we observed inhibition of transcription upon DSB induction, indicating that the presence of a DSB can inhibit transcription independently from its position relative to the gene (Supplementary Fig 5a,b). As a control, two distinct data sets of genes overlapping or adjacent (±2 Kb) to uncut *Asi*SI sites located in the promoter/TSS region or in the rest of the transcribed region, showed no substantial difference between induced and uninduced cells (Supplementary Fig. 5c,d).

In addition, to validate these conclusions, we induced a single DSB within the transcribed portion of a gene in two different cellular systems: (i) a human reporter cell line (DR-GFP U2OS) that allows for DSB generation 102 bp downstream of TSS via a doxycycline (Dox)-inducible I-SceI endonuclease^22^ and (ii) a mouse NIH3T3-derivative cell line also bearing the Lac-ISceI-Tet construct integrated in the genome^23^ in which we induced a single DSB targeting the *c-Myc* gene with a CRISPR-Cas9 approach (using two different RNA guides) in the last exon of *c-Myc*, located ∼280 bp upstream the 3′-UTR. Both approaches demonstrated that a DSB within a transcriptional unit inhibits the transcription of the respective gene (Supplementary Fig. 5e,f). Finally, as an additional control, a DSB induced in the NIH2/4 system by I-SceI endonuclease within an integrated construct (located away from the *c-Myc* gene locus) failed to downregulate *c-Myc*, demonstrating that the observed DSB-mediated *c-Myc* transcriptional repression is mediated in *cis* (Supplementary Fig. 5f).

### Transcription downregulation hinges on reduced RNA synthesis

**Figure 3:**
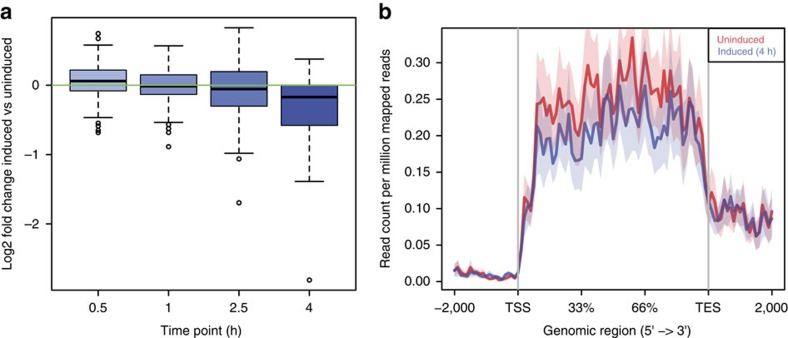
Bru-Seq data show downregulation at 4 h after DSB induction. (**a**) Boxplot representing log2 of the fold change of expression for genes (*n*=196) with cut *Asi*SI sites at different time points (30 min, 1, 2 and 4 h) after DSB induction. (**b**) Coverage profile plot representing the RPM of Bru-seq reads in induced (blue) and uninduced (red) samples for each genebody region. TES, transcription end site; TSS, transcription start site. Bold lines represent mean values, whereas semi-transparent shades around the mean curves represent the s.e.m. across the regions.

The conclusions described so far were based on studies of steady-state RNA levels; thus, they did not allow to distinguish whether the observed downregulation of gene expression was due to alterations in the rate of transcription initiation or RNA synthesis. To analyse changes in transcription rates without the confounding influence of pre-existing steady-state RNA, we decided to study only the transcriptional events occurring post DSB induction by performing a time-course assay based on 30 min pulse-labelling of RNA with Bru-seq^24^. By this approach, Bru-labelled RNAs are specifically captured and sequenced to reveal differential rates of RNA synthesis in the genome. We thus checked the levels of RNA synthesis by 30 min labelling at four time points (30 min, 1, 2 and 4 h after DSB induction) and compared the levels of transcription at DSB sites detected by BLISS in induced cells versus uninduced cells. Among the four different time points investigated, we observed a maximal decrease in transcription at 4 h post induction (Fig. 3a). We thus focused on the DE between induced and uninduced cells at the 4 h time point and computed a coverage profile plot comparing the RPM of the two samples distributed along the gene bodies. These analyses revealed an overall reduction of transcription following DSB induction (Fig. 3b), thus showing that the decrease of RNA levels observed in steady-state RNA-seq experiments depends on reduced rates of RNA synthesis upon DSB induction.

### CAGE data show decreased TSS activity

**Figure 4:**
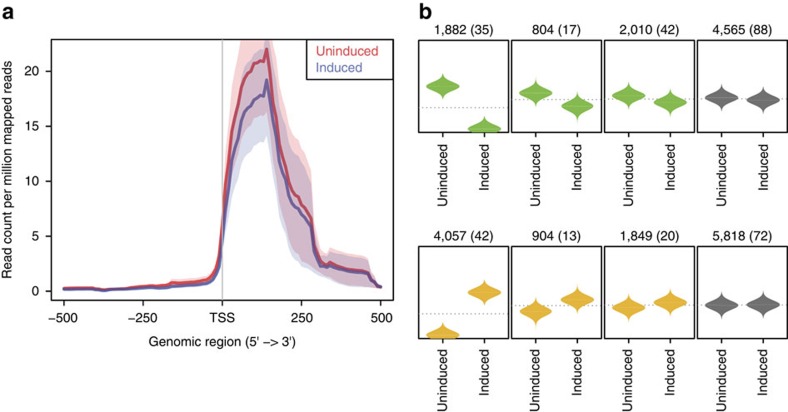
CAGE data show downregulation of TSS activity after the break. (**a**) Coverage profile plot representing the RPM of CAGE reads in induced (blue) and uninduced (red) samples at TSSs of genes (*n*=196) with cut *Asi*SI sites in a 500 bp window. TSS, transcription start site. Bold lines represent mean values, whereas semi-transparent shades around the mean curves represent the s.e.m. across the regions. (**b**) Promoter-centred expression profiling. Comparison of expression coverage profiles for TSS activities between uninduced and induced samples. Promoters are grouped into expression classes by applying *k*-means clustering. Each box represents one cluster with reported above the total number of promoters and in brackets the number of overlapping cut *Asi*SI sites. Individual beanplots show distribution of scaled normalized expression for uninduced and induced samples. Colour of the beanplots represents the different expression classes of TSS dynamics: downregulated TSSs in green; upregulated TSSs in yellow; nearly unchanged TSSs in grey. Individual CAGE experiments (uninduced or induced) are shown on *x* axis and scaled normalized expression on *y* axis.

We then focused our analyses on the effect of DSBs on transcription initiation. As the induced cut sites are enriched at TSS, we decided to map at single-nucleotide resolution the potential changes in transcription initiation activities upon *Asi*SI-induced DSB generation in both induced and uninduced cells and compare them. We performed CAGE, a technique that precisely maps and quantifies the steady-state abundance of the 5′-ends of RNA genome wide. We computed expression coverage profiles of the same set of 196 genes having an *Asi*SI site in their gene body or in their proximity (±2 Kb) as employed in RNA-seq analyses. We observed a clear downregulation of transcription initiation in induced versus uninduced cells, thus demonstrating that DSBs negatively affect TSS activity (Fig. 4a). In addition, to independently validate that downregulated genes are significantly enriched for induced DSBs, we performed clustering of CAGE-defined TSSs based on their expression profiles between induced and uninduced samples. This analysis revealed eight clusters that can be grouped in three major classes of TSS dynamics observed in the cell upon DSB generation: downregulated TSSs (Fig. 4b, green beanplots), upregulated TSSs (Fig. 4b, yellow beanplots) and nearly unchanged TSSs (Fig. 4b, grey beanplots). We thus calculated the number of cut AsiSI sites overlapping or proximal (up to ±2 Kb) to the TSSs belonging to the three major classes of TSS activities. Importantly, the first class (downregulated TSS activities in induced cells) was significantly enriched for cut *Asi*SI sites (*P*<0.05, Fisher’s exact test), confirming that the observed reduced transcription initiation rates are directly related to the presence of DSBs.

### Elongating RNAPII is reduced upon DSB induction

We then focused our analyses on the mechanism of transcriptional downregulation upon DSB induction. To use an independent approach, we mapped the levels of total and elongating (phosphorylated on serine 2) RNAPII by ChIP-seq, by the use of two different antibodies (see Methods). Despite unaltered levels of total RNAPII between induced and uninduced samples (Supplementary Fig. 6a,b), we observed a reduction of the elongating form of RNAPII comparing induced with uninduced samples (Supplementary Fig. 6c,d). These analyses revealed that DSB induction leads to reduction in the abundance of the elongating form of RNAPII at the DSB.

In sum our results from four independent approaches reveal that steady-state RNA levels are downregulated upon DSB induction, as determined by RNA-seq, and that this is the consequence of reduced transcription initiation, as demonstrated by CAGE analyses, and of reduced rates of RNA synthesis and elongation, as revealed by Bru-seq and RNAPII ChIP-seq experiments.

### Transcription inhibition depends on distance from the lesion

**Figure 5:**
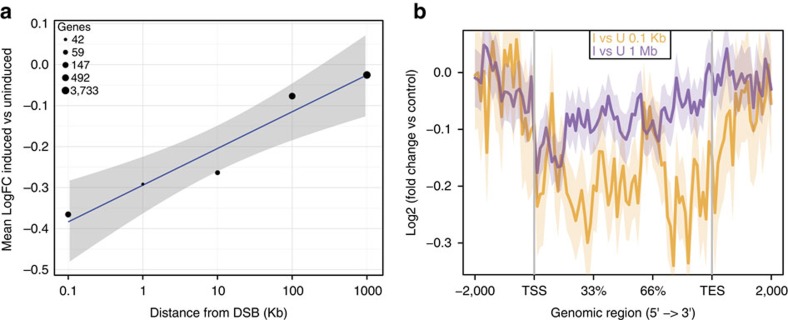
RNA-seq data show downregulation decreases upon increasing genomic distance. (**a**) Plot showing the mean log fold-change of expression in induced vs uninduced samples upon increasing genomic distance. Mean logarithm of the fold change was measured for each set of genes located at a certain distance from the break and normalized by the total number of sites checked. Distance from the DSB is reported in Kb on the *x* axis. The diameter of each circle represents the number of genes considered (as reported in figure legend). Blue line represents linear fit to the data and grey-shaded area the s.e. (**b**) Coverage profile plot representing the log fold change of RNA-seq reads in induced vs uninduced samples for genes located at 0–0.1 Kb from an *Asi*SI site (yellow) and 1 Mb from an *Asi*SI site (violet). TES, transcription end site; TSS, transcription start site. Bold lines represent mean values, whereas semi-transparent shades around the mean curves represent the s.e.m. across the regions.

The coupling of a fine map of DSBs generated upon *Asi*SI induction with RNA-seq data allowed us to test whether the transcriptional downregulation observed was sensitive to the distance of the affected gene from the DSB. We therefore tested the impact of DSBs on genes at increasing distances from the DSB (namely up to 0.1, 1, 100 and 1 Mb) by measuring the fold change of their expression in induced and uninduced samples. We observed a decrease of downregulation of gene expression upon increasing genomic distance (Fig. 5a), with the genes at 1 Mb of distance detectably unaffected (Fig. 5b). A parallel analysis using constant bins of 200 Kb reached similar conclusions, indicating that bin size does not influence the result (Supplementary Fig. 7). These results indicate that DSBs reduce transcription of damaged genes or genes immediately adjacent to them, whereas they do not significantly cause direct impact on the expression of more distal genes.

### Transcription downregulation is ATM dependent

**Figure 6:**
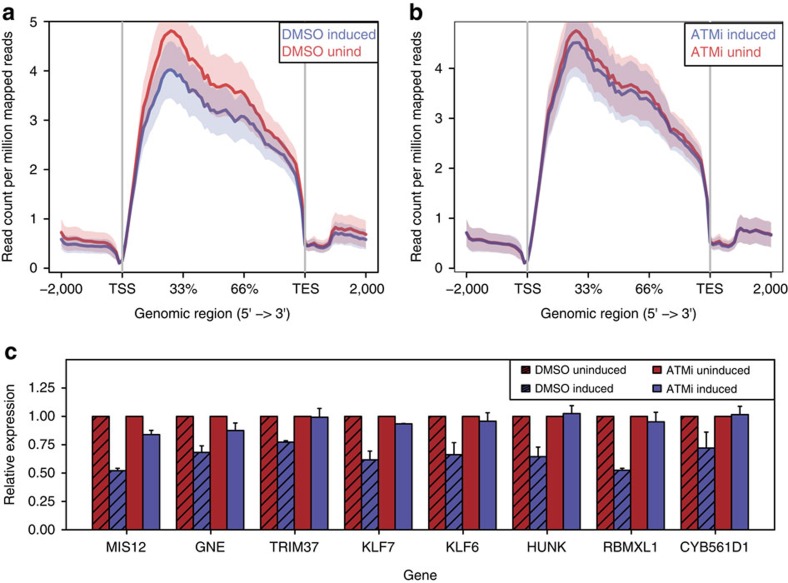
Transcription downregulation is ATM dependent. (**a**,**b**) Coverage profile plot representing the RPM of transcripts of genes with *Asi*SI sites cut according to BLISS in induced (blue) and uninduced (red) samples in DMSO (**a**) or ATMi (**b**) treated cells. TES, transcription end site; TSS, transcription start site. Bold lines represent mean values, whereas semi-transparent shades around the mean curves represent the s.e.m. across the regions. (**c**) qPCR validation of relative expression of eight randomly selected genes in induced (blue) versus uninduced (red) samples upon DMSO (bars with texture) or ATMi treatment. Shown is the mean of biological replicates (*n*=3). Error bars show s.e.m.

Our observation that transcription downregulation is inversely proportional to the increasing distance from the DSB raised an additional question, namely whether the observed downregulation is due to RNAPII activity being impeded by the presence of a DSB, or to the ensuing DDR. The latter was previously shown^5^ by the use of an integrated construct made of a reporter gene cloned downstream of a cluster of inducible DSBs. We thus asked whether ATM was involved in DSB-induced downregulation of transcription of endogenous genes in our genome-wide analysis of individual DSBs. We treated DIvA cells with a specific ATM kinase inhibitor (KU-60019) or its vehicle (dimethyl sulfoxide, DMSO) as a control, before DSB induction. As expected, ATM inhibition (ATMi) reduced *γ*H2AX accumulation (Supplementary Fig. 8a,b). We performed RNA-seq of DMSO-treated or ATMi-treated samples and observed the expected downregulation of transcription in DMSO-treated cells upon DSB induction (Fig. 6a), whereas this observed downregulation was prevented in ATMi-treated cells (Fig. 6b). RT–qPCR on eight randomly selected genes independently confirmed that ATM kinase activity is required for the transcriptional repression at individual genes (Fig. 6c). Thus, consistently with previous reports in an engineered construct^5^, we proved genome-wide and at endogenous genomic loci that ATM is involved in DSB-induced downregulation of transcription. Finally, to check whether ATM activity is required for transcriptional downregulation independently of the gene region where damage is induced, we investigated two distinct subsets of DSBs: those located upstream of a gene and those within the gene body. In both subsets, we observe DSB-induced downregulation. Importantly, downregulation was impaired in ATMi-treated cells (Supplementary Fig. 8c–f), indicating that ATM kinase activity is required to inhibit transcription also of those genes in which a DSB occurs within their transcriptional unit.

In conclusion, these results indicate that a DSB in the genome is not sufficient *per se*, to hinder RNAPII activity, but rather it initiates a signal transduction process depending on ATM kinase activity that inhibits transcription of the damaged gene and in the genes immediately adjacent to it.

## Discussion

The DNA damage field has recently benefited by novel technologies able to identify and map DSBs at single-nucleotide resolution^16^,^17^,^18^,^25^. A better understanding of the precise location of DSBs is critical to appreciate the dynamics of their impact on the genome (such as mutations and structural variations, recently described by genomic studies of various cancers) and on the transcriptome. Related to this, the increasing availability of approaches to study cellular RNA biology in a cell provides a perfect ally to couple the acquired knowledge on DNA damage generation with the study of the local and global transcriptional response.

Global alterations in gene expression, binding of transcription factors and activity of promoters and enhancers have been reported after DNA damage induction by ionizing radiation in breast cancer cells, thus regardless of the specific position of the DSBs^26^. Studies of the local transcriptional response to DNA damage instead showed apparently contradictory results, likely to be because of the study of different (exogenous versus endogenous) damaged genomic regions or of the different amounts and relative position of DSBs (cluster of DSBs in a single genomic locus versus individual DSBs across the genome)^4^,^5^,^7^.

Here we surveyed the impact of DSBs on pre-existing transcription, harnessing the power of next-generation sequencing and novel technologies to detect and localize on the genome at single-nucleotide resolution the DNA breaks generated in endogenous genomic regions and to characterize genome-wide the alterations of the transcriptional landscape.

In particular, we took advantage of BLISS, a novel nucleotide-resolution genome-wide DSB mapping technique that builds on our previously published method, BLESS^16^, providing higher sensitivity^15^. The coupling of BLISS with γH2AX ChIP-seq allowed us to further validate the presence and location of *Asi*SI cut sites. With our results we provide the most accurate and complete list of DSBs induced in a human cell line reported up to now, thus generating an important resource available for the scientific community for further studies.

By exploiting the ability to induce DSB at precisely mapped endogenous genomic sites, we studied the effects of DSBs on local transcription by examining differentially expressed genes by RNA-seq, rates of RNA synthesis by Bru-seq and transcription initiation by CAGE, and transcription elongation of RNAPII by ChIP-seq, and we validated these results by RT–qPCR. By means of these four independent techniques, we show that DSBs lead to the downregulation of steady-state RNA levels and they inhibit both RNA synthesis and transcription initiation and elongation events, unequivocally proving that the presence of a DSB in proximity to or within a transcribed region inhibits its transcription at several regulatory steps. Our results are in agreement with prior observations both in cultured cells and in an animal model reporting transcriptional repression in genes proximal to I-PpoI recognition sites, without however determining which sites were actually cut^6^.

Our observation that transcriptional repression is highest in proximity to the site of damage and decreases progressively away from it, suggests that the effect of DNA damage on transcription is spatially regulated. In agreement with previous reports^5^,^6^, we showed that this downregulation is dependent on ATM. However, although previously conclusions were drawn out of the study of exogenous loci upon induction of a cluster of DSBs^5^ or repetitive sequences without a precise mapping of the DSBs^6^, here we robustly showed that a single DSB within an endogenous and non-repetitive sequence is sufficient to trigger the downregulation of proximal or overlapping genes. This could be a mechanism of the cell to avoid the synthesis of defective transcripts that may prove dangerous for the cell. Interestingly, *Asi*SI breaks elicits a DDR response involving p53 (ref. 27).

By total RNAPII ChIP-seq experiments, we observed that RNAPII peaks often coincided with areas lacking γH2AX very close to the site of break (Supplementary Fig. 6e), as also previously reported^7^, showing that even if transcription is affected by DSBs, RNAPII can still be found in regions where the DDR signalling spreads. This is compatible with two different scenarios: a stalling of the elongating transcriptional machinery and/or a *de novo* transcription upon break. Interestingly, the latter is consistent with the biogenesis of novel DSB-dependent transcripts, which can in turn be processed into DDRNAs or DSB-induced RNAs, important for DDR foci formation and DNA repair^8^,^9^,^28^. Our recently developed target enrichment approach that allowed robust detection of low abundance DDRNAs at dysfunctional telomeres^28^ may be of use to inspect the synthesis and biogenesis of novel DSB-induced transcripts in a genomic context where pre-existing transcripts and/or pervasive transcription of the genome^29^ may blur the presence of newly synthesized RNA molecules at the site of break.

Finally, given the recent advances in next-generation sequencing technology and its reduced costs, we foresee that our approach will be applied to different systems such as DSB-prone conditions or cancer cells and set the basis for a high-resolution mapping of genome and transcriptome alterations induced by different sources of DNA damage.

## Methods

### Cell culture

DIvA (*Asi*SI-ER-U20S^7^) cells (kind gift from Gaëlle Legube) were cultured in DMEM medium (Gibco) without phenol red, supplemented with glutamine, pyruvate, HEPES and 10% fetal bovine serum (FBS, Euroclone). Cells were grown at 37 °C under a humidified atmosphere with 5% CO_2_ and selected on a semi regular basis with puromycin at a final concentration of 1 μg ml^−1^. For *Asi*SI-dependent DSB induction, cells were treated with 300 nM 4OHT (Sigma; H7904) for 4 h. Cells were treated with DMSO or ATMi (KU-60019; Sigma SML1416) at a final concentration of 10 μM, simultaneously with 4OHT treatment.

Dox-inducible I-SceI/DRGFP cells (TRI-DR-U2OS)^22^ (kind gift of Philipp Oberdoerffer) were cultured in DMEM medium with 10% FBS at 37 °C in the presence of 5% CO2. I-SceI expression was induced by adding 5 μg ml^−1^ Dox for 12 h.

NIH2/4 cells, a NIH3T3-derived cell line bearing the Lac-ISceI-Tet construct integrated in the genome^23^ were grown in DMEM (Lonza), supplemented with 10% FBS Tetracycline tested, 1% L-glutamine, 1% penicillin/streptomycin and hygromycin (400 μg ml^−1^).

Vectors expressing the restriction enzyme I-SceI (kind gift from E. Soutoglou), CRISPR-Cas9 sgRNA#8 (5′-ACACGGAGGAAAACGACAAG-3′) or CRISPR-Cas9 sgRNA#9 (5′-CAGACACGGAGGAAAACGAC-3′) targeting *c-Myc* gene (kind gift from B. Amati) or an empty vector control were transfected at the concentration of 2 μg in NIH2/4 cells by Lipofectamine 2000 (Life Technologies), according to the manufacturer’s protocol.

All cell lines used in this study were tested negative for mycoplasma contaminations.

### Immunofluorescence

Cells were grown on glass coverslips, washed twice with ice-cold PBS and treated with ice-cold permeabilization buffer (PBS with 0.4% Triton X-100) before fixation with 4% paraformaldehyde at room temperature (RT) for 10 min. After two washes in PBS, cells were incubated for 1 h in blocking solution (PBG, 0.5% BSA, 0.2% gelatin from cold water fish skin in PBS) and then stained with primary antibodies diluted in PBG overnight at 4 °C in a humidified chamber. Cells were washed 3 times for 5 min with PBG and incubated with secondary antibodies diluted in PBG for 1 h at RT in a dark humidified chamber. Cells were washed twice for 5 min with PBG, twice for 5 min with PBS and incubated with 4′-6-Diamidino-2-phenylindole (DAPI, 0.2 μg ml^−1^, Sigma-Aldrich) for 2 min at RT. Cells were briefly washed with PBS and water, and coverslips were then mounted with Aqua Poly/Mount (Polysciences) mounting medium and let dry overnight at room temperature. Images were acquired with widefield Olympus Biosystems Microscope BX71 and the analySIS or the MetaMorph software (Soft Imaging System GmbH). Comparative immunofluorescence analyses were performed in parallel with identical acquisition parameters. Number of foci per cell were analysed by the imaging software CellProfiler^30^.

### Antibodies

Anti-γH2AX (rabbit, Cell Signaling (20E3), 1:2000 for immunofluorescence).

Anti-gamma H2A.X (phospho S139) antibody (ABCAM, AB2893, 2 μg for ChIP).

Anti-RNAPII CTD repeat YSPTSPS (phospho S2) antibody (Rabbit, ABCAM, AB5095, 2 μg for ChIP).

Anti-RNAPII antibody, clone CTD4H8, (mouse, MILLIPORE, 05-623, 2 μg for ChIP).

### BLISS linker preparation

The single-strand oligonucleotides listed in Supplementary Table 3 were annealed in purified water, at a final concentration of 10 μM. The oligonucleotides mix was heated to 95 °C for 5 min. Tubes were removed from the heat source and slowly cooled to room temperature.

### BLISS

BLISS was performed on DIvA (*Asi*SI-ER-U20S) cells mock treated or induced with 4-OHT using one coverslip per condition (11 millimiters), containing ∼30.000 adherent cells. Briefly, 4 h after induction, cells were washed with PBS and fixed in 4% paraformaldehyde for 10 min at room temperature. After washing, cells were lysed and submitted to *in situ* DNA ends blunting (with Quick Blunting kit, NEB), followed by *in situ* DNA ends ligation with BLISS linker (Supplementary Table 3). After washing, genomic DNA was extracted and sonicated with Covaris S220 (10% duty factor, 175 W peak incident power, 200 Cycles/burst, 105s) to obtain a pool of 300 bp fragments. Afterwards, fragmented DNA was *in vitro* transcribed using MessageAmpII kit (Ambion) for 14 h at 37 °C. After RNA purification and ligation of the 3′-Illumina adapter, the RNA was reverse transcribed. The final step of library indexing and amplification was performed using the Illumina TruSeq Small RNA Library Prep Kit.

### Preparation of ChIP DNA libraries

For RNAPII ChIP-seq experiments, cells were crosslinked for 5.5 min at room temperature with Fixation Buffer (1% formaldehyde, 100 mM NaCl, 1 mM EDTA, 0.5 mM EGTA, 50 mM HEPES pH 7.4). Crosslinking was quenched by addition of glycine (125 mM). Fixed cells were rinsed twice in 1 × PBS, collected by scraping and centrifuged at 1,840 × g for 5 min at 4 °C. Pellets were re-suspended in cold B1 Buffer (0.25% Triton X-100, 1 mM EDTA, 0.5 mM EGTA, 10 mM Tris pH 8; Proteases inhibitors (Roche); Microcystin (Enzo Life Sciences)) by mixing for 10 min on a rotating wheel at 4 °C and then centrifuged at 1,840 × g for 5 min at 4 °C. The same steps were repeated with cold Buffer B2 (200 mM NaCl, 1 mM EDTA, 0.5 mM EGTA, 10 mM Tris pH 8; Proteases inhibitors (Roche); Microcystin (Enzo Life Sciences)). Finally, pellets were re-suspended in cold Buffer B3 (TE 1 × ; EGTA 0.5 mM) in a suitable volume. Pellets were sonicated using a Focused-Ultrasonicator Covaris (duty: 5.0, PIP: 140, cycles: 200, amplitude: 0, velocity: 0, dwell: 0, microTUBEs with AFA fibre). Sonicated chromatin was diluted in RIPA buffer (1% TritonX-100, 0.1% Na- Deoxycholate, 0.1% SDS, 64 mM NaCl, 10 mM Tris HCl pH 8.0) to give a concentration of ∼100 μg in 400 μl per ChIP. Samples were pre-cleared for 2 h, rotating at 4 °C, with 20 μl of magnetic beads (Dynabeads Protein G, LifeTechnologies) per ChIP. Samples were then incubated overnight rotating at 4 °C with specific antibodies (see Antibodies section for a complete list) or no antibody (mock). The bound material was recovered by 2 h incubation with 20 μl of magnetic beads per ChIP. Beads were then washed, rotating at 4 °C for 10 min, four times in RIPA buffer, once in LiCl buffer (250 mM LiCl, 0.5% NP-40, 0.5% Na Deoxycholate, 1 mM EDTA, 10 mM Tris-HCl pH 8) and finally in 1 × TE. ChIPed material was eluted by 15 min incubation at 65 °C with 150 μl Elution Buffer (1% SDS, 10 mM EDTA, 50 mM Tris HCl pH 8). Samples were reverse-crosslinked by incubation with proteinase K (Invitrogen) at 37 °C for 5 h and then at 65 °C overnight. DNA was cleaned up by QIAquick PCR purification column (Qiagen), according to the manufacturer’s instructions, and eluted in 30 μl of elution buffer.

For γH2AX ChIP-seq experiments, *in vivo* crosslinking, chromatin purification and immunoprecipitations were carried out as previously described^31^. Briefly, cells were crosslinked for 7 min at 37 °C with the Fixation Buffer and treated as above. Pellets were re-suspended in cold Buffer B3+SDS (TE 1 × , EGTA 0.5 mM, 0.1% SDS) in a suitable volume (20 × 10^6^ of starting cells in 1 ml). Samples were aliquoted in 0.5 ml Bioruptor Plus Microtubes (C30010013, Diagenode) for sonication with Bioruptor Plus (Diagenode). Sonication was performed with high power mode for 30 cycles (sonication cycle: 30 s ON, 30 s OFF) to obtain fragments of 200–500 bp. Sonicated chromatin was diluted in RIPA buffer and processed as above.

### RNA extraction and library preparation

For total RNA-seq and CAGE we used the *mir*Vana microRNA Isolation Kit (Ambion) according to the manufacturer’s instructions for the collection of total RNA. For total RNA-seq, before library preparation, 2.5–5 μg total RNA samples (longer than 200 bp) were treated to remove ribosomal RNAs by the Ribo-Zero Gold kit (Epicentre). The ribosomal RNA-removed samples were then used for paired-end RNA-seq library preparation by the ScriptSeq v2 RNA-Seq Kit (Epicentre), according to the manufacturer’s protocol. For total RNA-seq of DMSO- and ATMi-treated cells, total RNA was isolated using the RNeasy Kit (QIAGEN), according to the manufacturer’s instructions, and strand-specific library preparation was performed using Illumina TruSeq kit. CAGE libraries were prepared using 2.5–5 μg of total RNA material (longer than 200 bp) following the protocol developed by Carninci lab^32^. Briefly, the 5′-cap structure of RNA molecules was oxidized by NaIO_4_ and labelled by a long-arm biotin hydrazide, followed by enzymatic digestion, priming and first-strand complementary DNA synthesis reactions. The biotinylated cDNA products were captured by streptavidin coated magnetic beads, washed, recovered and were then ligated to barcoded linkers for second strand cDNA synthesis. The double-strand cDNA products were cleaved by EcoP15I enzyme to generate 27nt CAGE tags, which were then ligated to 3′-linker and PCR amplified to generate final libraries. For Bru-seq experiment, cells were incubated at different times after *Asi*SI induction in 2 mM Bru for 30 min and total RNA was isolated using TRIzol (Invitrogen), according to the manufacturer’s instructions, and strand-specific libraries were prepared with the Illumina TruSeq Kit.

### Quantitative RT–PCR analysis

Total RNA was isolated from cells using the *mir*Vana microRNA Isolation Kit (Ambion) or the RNeasy Kit (QIAGEN), according to the manufacturer’s instructions. A 10 μg quantity of total RNA was treated with DNase (Ambion) for 20 min to remove any potential residual genomic DNA contamination. One microgram of total RNA was reverse-transcribed with the Superscript III First Strand cDNA synthesis kit (Invitrogen) with random examers. Expression of genes near the *Asi*SI cut sites in induced (cut) and uninduced (uncut) cells was evaluated by qPCR using QuantiTect SYBR green reagent (Qiagen). Real-time qPCR reactions were performed on a Roche Lightcycler 480 II Sequence Detection System. For each reaction, 20 ng of cDNA were used and Ribosomal protein P0 or β-2-Microglobulin were used as a control gene for normalization.

TRI-DR-U2OS RNA was extracted by using the Maxwell RSC simplyRNA Kits, according to the manufacturer’s instructions. RNA was reverse transcribed with SuperScript VILO Reverse Transcriptase (Life Technologies). RT–qPCR was performed on a Roche LightCycler 480 machine using Roche SYBR. Primers GFP-FW and GFP-RV were used to detect the green fluorescent protein (GFP) start transcript and ribosomal protein P0 was used as normalizer.

NIH2/4 RNA was isolated 24 h later using Maxwell RSC simply RNA kit (Promega), according to the manufacturer’s instructions. cDNA was generated using the SuperScript VILO Reverse Transcriptase (Life Technologies). SYBR Green-based RT–qPCR experiments were performed on a Roche LightCycler 480 sequence detection system using Roche SYBR. Ribosomal protein P0 was used as housekeeper for normalization.

The list of primers used for qPCR analysis is reported in Supplementary Table 4.

### *Asi*SI *in silico* prediction

*In silico* digestion of the GRCh37/hg19 assembly was performed using the program Restrict from the EMBOSS package (http://emboss.sourceforge.net) to compute a map of *Asi*SI restriction sites (5′-GCGATCGC-3′) present on the human genome.

### BLISS sequencing and data analysis

Library quality and quantity was assessed on the 2100 Bioanalyzer (Agilent) using the High Sensitivity DNA kit (Agilent). Clusters were generated on the Illumina flow cell using the automatic cBot station and the TruSeq PE Cluster Kit v3-cBot-HS. Sequencing was carried out on Illumina HiSeq 2000 using the TruSeq SBS Kit v3-HS chemistry.

Paired-end sequencing reads from each sample were mapped to the human genome (GRCh37/hg19) using BWA^33^. At most, one mismatch per read was allowed and duplicated reads were removed using rmdup of SAMtools^34^. All reads uniquely mapping were scanned for the presence of the proximal linker barcode with *ad hoc* scripts written in bash and R languages. Finally, all reads within 100 bp of the *Asi*SI sites identified by BEDtools intersect^35^ were considered on target and retained for further analysis.

To rank the cutting efficiency at individual *Asi*SI sites, a four steps normalization procedure was applied:

- for each window of ±100 bp surrounding an *Asi*SI site, the fraction of bases that were overlapped by at least one paired-end read (covered fraction, cf) was calculated using BEDtools coverage^35^;
- the number of reads originating inside the ±100 bp window (read coverage, rc) covering each position was calculated using SAMtools mpileup^34^; 
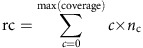
- a local normalization—ln—was applied for each *Asi*SI site, where the read coverage was normalized by the covered fraction. 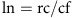
- For *Asi*SI each site, the ln counts were normalized by the median ln counts of all inspected sites to perform a global normalization—gn.

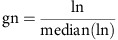


To identify the *Asi*SI sites that where cut upon *Asi*SI induction, we compared the cutting efficiency at individual *Asi*SI sites in induced and uninduced samples with a two step procedure:

- a quantile normalization-*qn*-was applied in order to make the two distributions identical in statistical properties. In this normalization, the highest entry in the induced distribution takes the value of the highest entry in the uninduced distribution and so on. *qn* counts were thus obtained for each site.
- For each site, the ratio and the log2ratio of induced and uninduced *qn* counts were finally calculated and used for the final ranking.

### Selection of an *Asi*SI site validated subset

To assess the performance of BLISS, a subset of *Asi*SI sites was selected according to their evidence of being detectably cut as reported in literature^7^,^19^,^20^. In particular, 15 *Asi*SI sites reported to be cut by ligation-mediated purification^36^ were used as positive controls for BLISS, whereas three additional *Asi*SI sites, reported not to be cut by the same technique, were used as negative controls (Supplementary Table 2).

According to ligation-mediated purification, a biotinylated linker with cohesive ends complementary to the *Asi*SI cut site is ligated to the cleaved sites. After strepdavidin purification of the labelled sequences, qPCR is performed to detect enrichment of specific sequences. This low-throughput technique is able to detect only clean-cut, not resected, *Asi*SI-induced DSBs, an ideal benchmark to test BLISS efficiency.

In particular, 15 *Asi*SI sites reported to be cut were used as positive controls and 3 additional *Asi*SI sites, reported not to be cut, were used as negative controls (Supplementary Table 2).

### ChIP-seq data analysis

For γH2AX ChIP-seq experiments, clusters were generated by ‘connecting’ γH2AX peaks (windows of 250 bp or multiples thereof in which γH2AX ChIP-seq signal is significantly—*z*-score>3—enriched over the background) if their distance is less than a ‘window size’ *n*. In other words, two adjacent γH2AX peaks A and B, whose midpoints are *m* nucleotides apart on the same chromosome, will be collapsed into a single cluster—covering peaks A, B and the distance in between—if *m* is smaller than or equal to *n*. The cluster may be further extended if there is another γH2AX peak C equal to or less than *n* nucleotides away from the midpoint of peaks A or B (and so on). If, for a given γH2AX peak C, the distance to its nearest neighbour peak is bigger than *n* (or if there were no other peak on that chromosome), the resulting cluster will be equivalent to C itself. Finally, we introduced a γH2AX rank score as an indicator of the likelihood of a given site to be in a cleaved state upon *Asi*SI induction.

For RNAPII ChIP-seq experiments, preliminary sequencing quality assessment was performed using FastQC (http://www.bioinformatics.babraham.ac.uk/projects/fastqc/). The samples passing the literature quality standards were aligned on the human genome (GRCh37/hg19) using BWA^37^. To maintain the collinearity between the read signal and the protein occupancy on the genome, multiple-matching reads were eliminated using *ad hoc* SAMtools^34^ and UNIX shell integrated scripts. Visualization and integration of the aligned data set was obtained with ngs.plot^38^.

### Coverage profile plots

Coverage profile plots at TSSs or genebody regions were computed using the ngs.plot package, an R-based data mining and visualization tool for next generation sequencing data^38^. This tool is based on two steps of normalization; in the first step of length normalization regions of variable sizes are equalized. In the second step, the vectors are normalized against the corresponding library size to generate the so-called RPM values that allow two next generation sequencing samples to be compared regardless of differences in sequencing depth. Ngs.plot uses the exon coordinates for each transcript (annotation sources: Ensembl V75.0; hg19 (GRCh37); homo_sapiens) to concatenate the coverage vectors for exons in order to simulate RNA splicing *in silico*.

### RNA-seq data analysis

The reads for RNA-seq experiment were aligned to the GRCh37/hg19 assembly human reference genome using the STAR aligner^39^. For differential testing, we used the package DESeq2 (ref. 40), in particular we used the regularized-logarithm transformation that stabilizes the variance across the mean. For genes with high counts, the regularized-logarithm transformation will give similar result to the ordinary log2 transformation of normalized counts. For genes with lower counts, however, the values are shrunken towards the genes’ averages across all samples.

### Bru-seq data analysis

Bru-seq was performed and analysed as previously reported^24^. Briefly, reads were aligned to the human ribosomal DNA complete repeating unit (U13369.1) using Bowtie (v0.12.8) and the reads that remained unaligned were mapped to the human genome build hg19/GRCh37 TopHat (v1.4.1). Bru-seq data from induced samples were compared with uninduced samples and fold differences determined.

### CAGE data analysis

CAGE data were analysed with the CAGEr package^41^. Briefly, after bam file preprocessing and quality (mappingQ≥20) filtering, CAGE tags were normalized and TSSs distance-based clustering (20 bp distance) was performed. Then, aggregate tag clusters across all CAGE data sets were computed and finally promoters were grouped into expression classes by applying *k*-means unsupervised clustering algorithm.

### Data availability

The data sets generated during the current study are available in the GEO (Gene Expression Omnibus) repository, GSE97589.

## Additional information

**How to cite this article:** Iannelli, F. *et al*. A damaged genome’s transcriptional landscape through multilayered expression profiling around *in situ*-mapped DNA double-strand breaks. *Nat. Commun.* **8**, 15656 doi: 10.1038/ncomms15656 (2017).

**Publisher’s note:** Springer Nature remains neutral with regard to jurisdictional claims in published maps and institutional affiliations.

## Supplementary Material

Supplementary Information Supplementary figures, supplementary tables, supplementary note and supplementary references.
