# Supplementary material for: A damaged genome’s transcriptional landscape through multilayered expression profiling around in situ-mapped DNA double-strand breaks
Source: Nat Commun. 2017 May 31;8:15656. doi: 10.1038/ncomms15656 (PMC5499205; doi:10.1038/ncomms15656)
Supplement: Supplementary Information — Supplementary figures, supplementary tables, supplementary note and supplementary references. [file ncomms15656-s1.pdf]

Supplementary Note 1

Analysis of AsiSI sites not detected by BLISS

We manually inspected the 26 sites detected only by γH2AX ChIP-seq (Supplementary Figure 3a), for the presence of specific features that could indicate different detectability of these sites. First, we checked for the genic location of the AsiSI site (Supplementary Figure 3b), and we did not detect any distinct feature that could justify their different detectability.

We then evaluated the expression levels of genes in the absence of DSB, analyzing RNAseq levels in the uninduced sample in three subsets of genes: the ones next to the 74 sites detected by both BLISS and γH2AX ChIP-seq, those next to the 26 γH2AX ChIP-seq only sites, and those next to the 140 BLISS only sites (Supplementary Figure 3a). We observed that sites detected by both BLISS and γH2AX ChIP-seq where the ones next to genes with significantly higher levels of transcription, with respect to the genes bearing the sites detected only by γH2AX ChIP-seq and only by BLISS (Supplementary Figure 3c). Since the presence of active transcription can be an indication of an open chromatin state that makes AsiSI sites particularly accessible to enzyme cleavage, the results indicate that AsiSI sites detected by both BLISS and γH2AX approaches are the ones with DSBs induced with the highest frequency. Moreover, since only 4 out of 26 (15%, Supplementary Figure 3d) γH2AX ChIP-seq specific sites were detected consistently by other approaches[1-3] while a significantly higher amount of sites detected by both BLISS and γH2AX ChIP-seq were instead confirmed by other approaches[1-3] (56 out of 74, 76%, P=0.004742, Pearson's Chi-squared test, Supplementary Figure 3e), we believe that the majority of sites missed by BLISS might be poorly cut and thus borderline affected in different experiments.

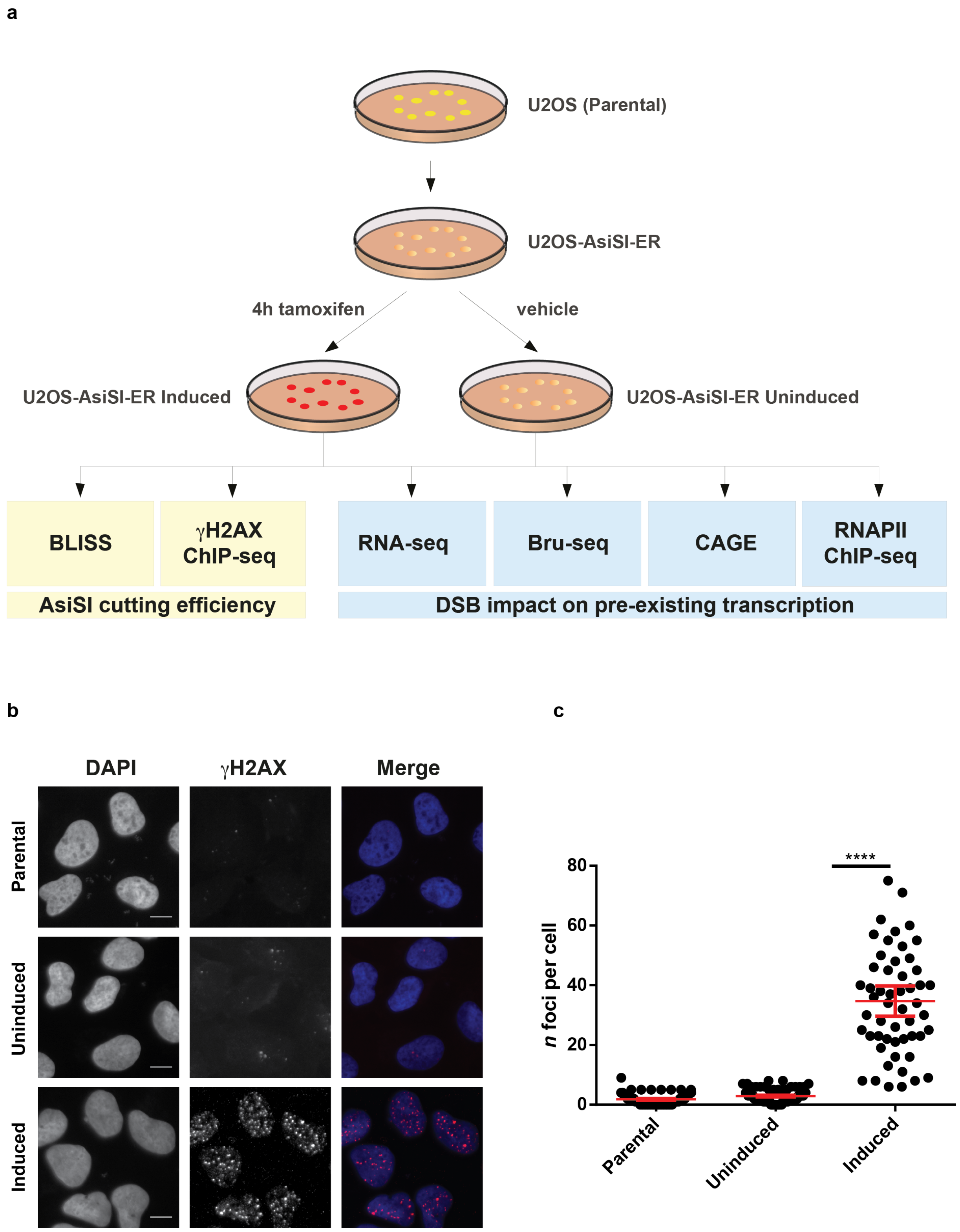

a
U2OS (Parental)
U2OS-AsiSI-ER
4h tamoxifen
vehicle
U2OS-AsiSI-ER Induced
U2OS-AsiSI-ER Uninduced
BLISS
γH2AX ChIP-seq
RNA-seq
Bru-seq
CAGE
RNAPII ChIP-seq
AsiSI cutting efficiency
DSB impact on pre-existing transcription
b
DAPI
γH2AX
Merge
Parental
Uninduced
Induced
c
n foci per cell
80
60
40
20
0
****
Parental
Uninduced
Induced

Supplementary Figure 1. (**a**) Scheme of the experimental approach used. See main text for description. (**b,c**) Representative images (**b**) and quantification analyses (**c**) of immunofluorescence for γH2AX in DIvA (AsiSI-ER-U2OS) cells, induced with 4OHT for 4 hours, uninduced (mock treated) or parental (U2OS cells lacking the AsiSI-ER-U20S construct); DNA was stained by DAPI. Scale bars: 10µm. DNA damage is induced by the translocation of *Asi*SI in the nucleus.

a

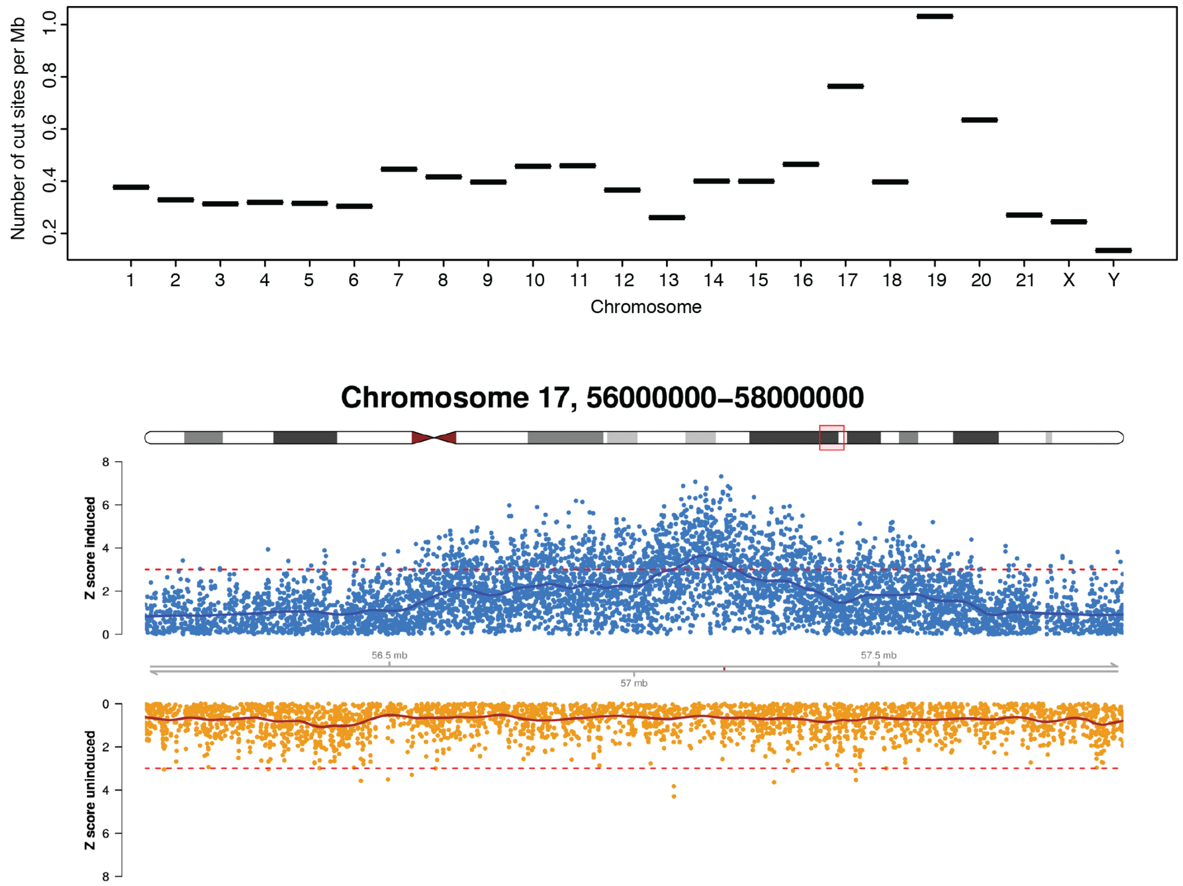

Supplementary Figure 2. (**a**) Number of predicted *Asi*SI sites upon *in silico* digestion of the GRCh37/hg19 human genome assembly. The number of sites has been normalized by the length of each chromosome. (**b**) Top-ranked *Asi*SI-induced cleavage site. The *Asi*SI recognition site with the highest γH2AX rank score is depicted. The panel represents a 20-fold zoom out, centered on the ~100 Kb window across which individual z-scores were averaged to calculate the score. The LOESS curves represent average z-scores. Absence of dots indicates that no positive z-scores were calculated for the respective windows (no or negative enrichment over background).

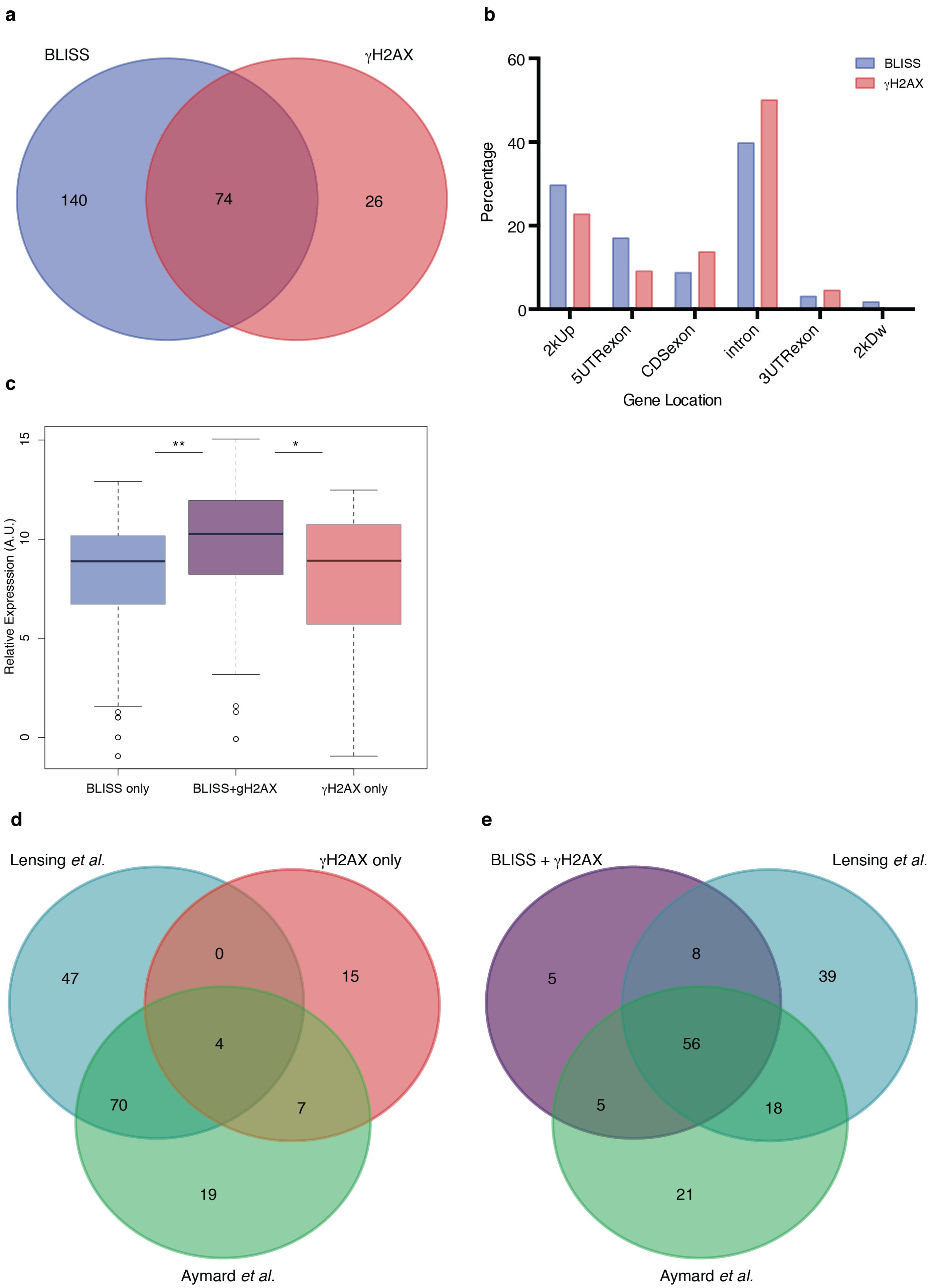

Supplementary Figure 3. (**a**) Venn diagram plot showing intersection of the 214 sites detected by BLISS (blue) and the 100 top sites detected according to γH2AX ChIP-seq (red).

(**b**) Percentage of genic regions upstream of a TSS (up to 2 Kb, 2KUp), 5' UTR exons (5UTRexon), Coding Sequence Exons (CDSexon), 3' UTR exons (3UTRexon), and downstream of a TES (up to 2 Kb, 2KDw), having an AsiSI site detected by BLISS (blue), or detected only by γH2AX ChIP-seq (red). (**c**) Boxplot representing the relative expression of transcripts in uninduced sample overlapping or adjacent (± 2 Kb) to the 140 BLISS only sites (blue), to the 74 sites detected by both BLISS and γH2AX ChIP-seq (purple), and to the 26 γH2AX ChIP-seq only sites (red). * = P<0.05, Wilcoxon test; ** = P<0.01, Wilcoxon test. (**d**) Venn diagram plot showing intersection of the 26 sites detected by γH2AX ChIP-seq and not by BLISS (red), by a previously published ChIP-seq experiment[2] (green) and by a recently published approach based on DSBCapture[3] (light blue). (**e**) Venn diagram plot showing intersection of the 74 sites detected by both BLISS and γH2AX ChIP-seq (purple), by a previously published ChIP-seq experiment[2] (green) and by DSBCapture[3] (light blue).

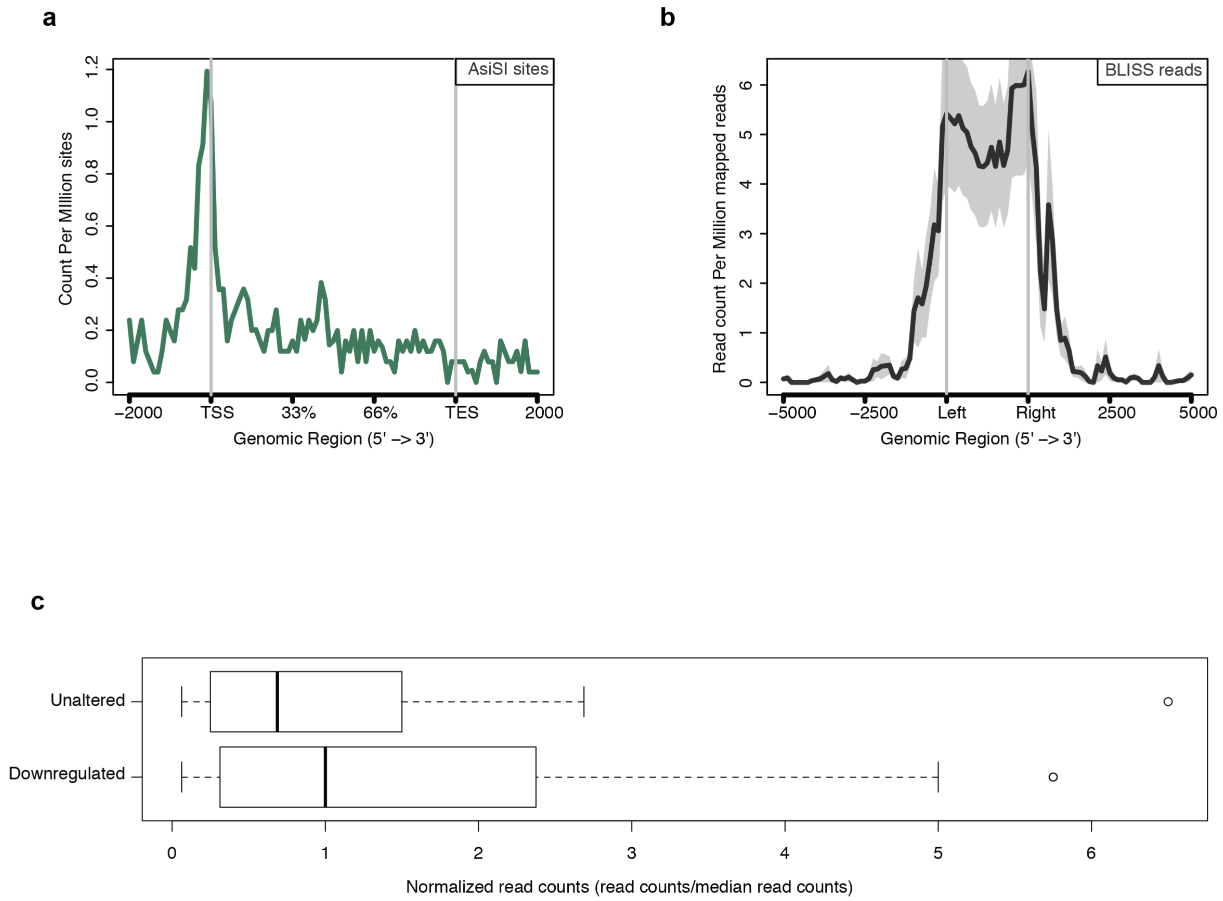

Supplementary Figure 4. (**a**) Coverage profile plot representing the *Asi*SI sites predicted upon *in silico* digestion of the GRCh37/hg19 human genome assembly. TSS = transcription start site, TES = transcription end site. (**b**) Coverage profile plot representing the reads per million mapped reads (RPM) of *Asi*SI sites detected by BLISS for each DNase I hypersensitive site (DHS) region. Left = left border of DHS site, Right = right border of DHS site. Bold lines represent mean value, while the semi-transparent shades around the mean curve represent the standard error of mean (SEM). (**c**) Boxplot representing BLISS normalized read counts (read counts/median read counts), for AsiSI sites located proximal to/in genes with no transcriptional alteration (Unaltered) and AsiSI sites proximal to/in genes showing transcription downregulation (Downregulated).

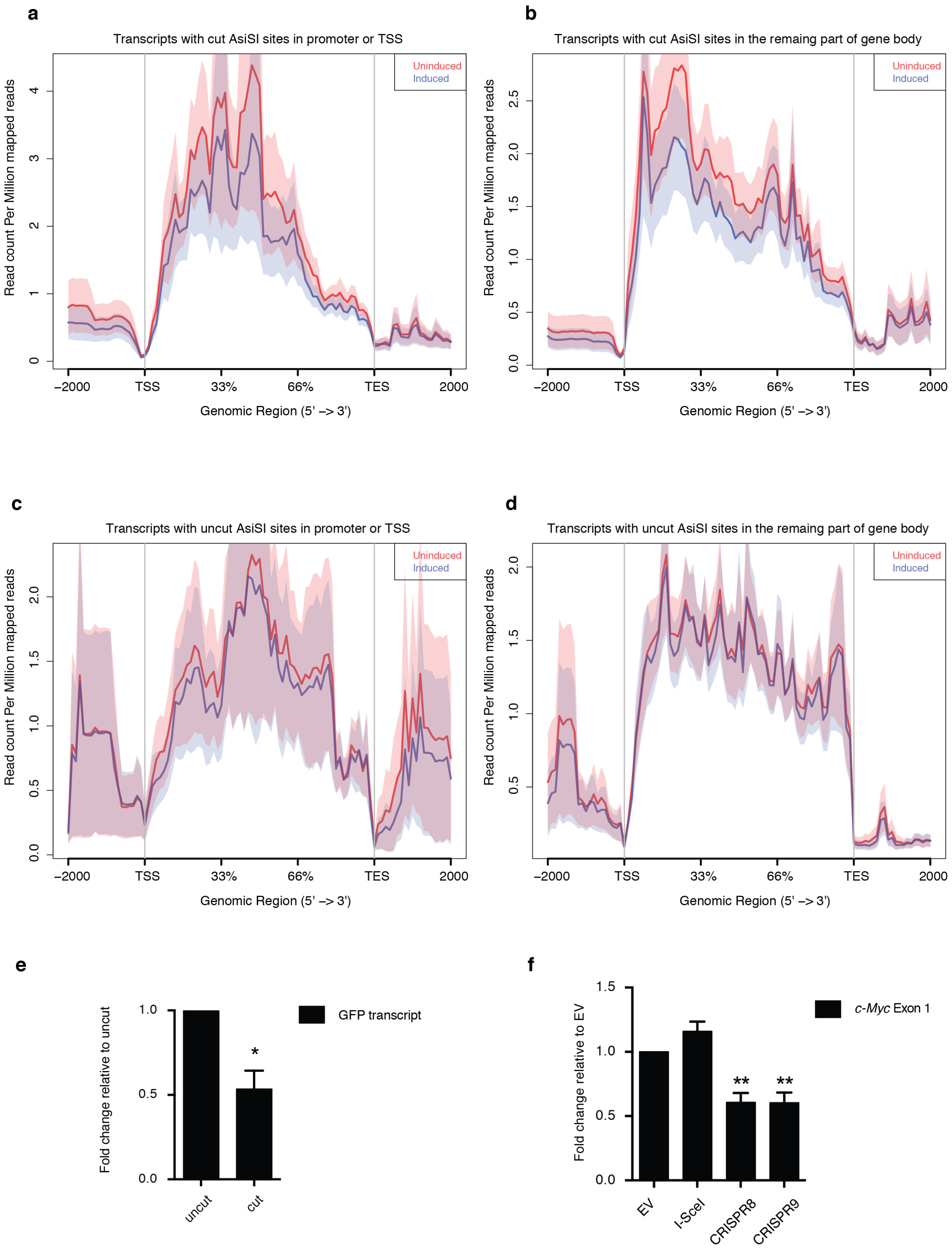

Supplementary Figure 5. (**a**) Coverage profile plot representing the RPM in induced (blue) and uninduced (red) samples of transcripts overlapping or adjacent (± 2 Kb) to cut *Asi*SI sites located in the promoter region or in proximity to the TSS. TSS = transcription start site, TES

= transcription end site. Bold lines represent mean value, the semi-transparent shades around the mean curve represent the SEM. (**b**) Coverage profile plot representing the RPM in induced (blue) and uninduced (red) samples of transcripts overlapping or adjacent (± 2 Kb) to cut *Asi*SI sites located in the remaining part of the gene body (*i.e.* introns, coding exons or 3' UTR exons). (**c**) Coverage profile plot representing the RPM in induced (blue) and uninduced (red) samples of transcripts overlapping or adjacent (± 2 Kb) to uncut *Asi*SI sites located in the promoter region or in proximity to the TSS. (**d**) Coverage profile plot representing the RPM in induced (blue) and uninduced (red) samples of transcripts overlapping or adjacent (± 2 Kb) to uncut *Asi*SI sites located in the remaining part of the gene body (*i.e.* introns, coding exons or 3' UTR exons). (**e**) Fold change measured by RT-qPCR of GFP transcript in a human reporter cell line (DR-GFP U2OS)[4] upon DSB generation via a doxycycline-inducible I-SceI endonuclease. The bar plot shows the relative levels of the indicated RNA sets upon cut. Uncut sample was used as reference. Error bars indicate SEM (n=3). (**f**) Fold change measured by RT-qPCR of *c-Myc* gene transcript in NIH2/4 cells (NIH3T3-derivative cell line)[5] upon DSB induced with a CRISPR-Cas9 approach (using two different RNA guides CRISPR8 and CRISPR9) or by I-SceI endonuclease within the Lac-ISceI-Tet integrated construct (I-SceI). The bar plot shows the relative levels of the indicated RNA sets upon cut. Uncut sample (empty vector, EV) was used as reference. Error bars indicate SEM (n=3).

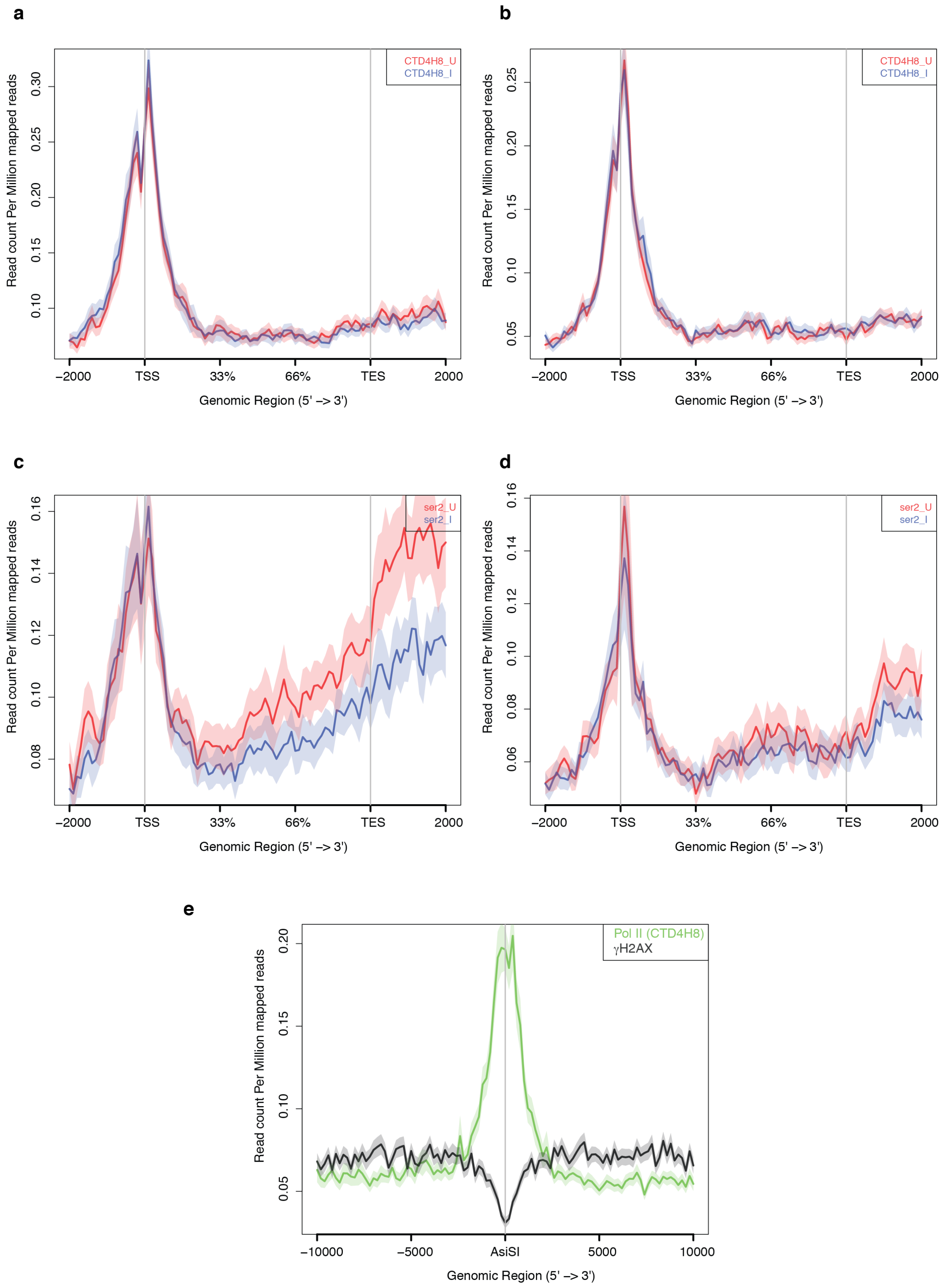

Supplementary Figure 6. (**a,b**) Coverage profile plot representing the RPM in induced (blue) and uninduced (red) samples of ChIP-seq reads for total RNAPII in genes overlapping or adjacent (± 2 Kb) to cut (**a**) and uncut (**b**) *Asi*SI sites, the semi-transparent shades around

the mean curve represent the SEM. (**c,d**) Coverage profile plot representing the RPM in induced (blue) and uninduced (red) samples of ChIP-seq reads for elongating RNAPII in genes overlapping or adjacent (± 2 Kb)) to cut (**c**) and uncut (**d**) *Asi*SI sites. (**e**) Coverage profile plot representing the RPM for total RNAPII ChIP-seq (green) and γH2AX ChIP-seq (black) in induced samples in a 10 Kb window surrounding *Asi*SI sites.

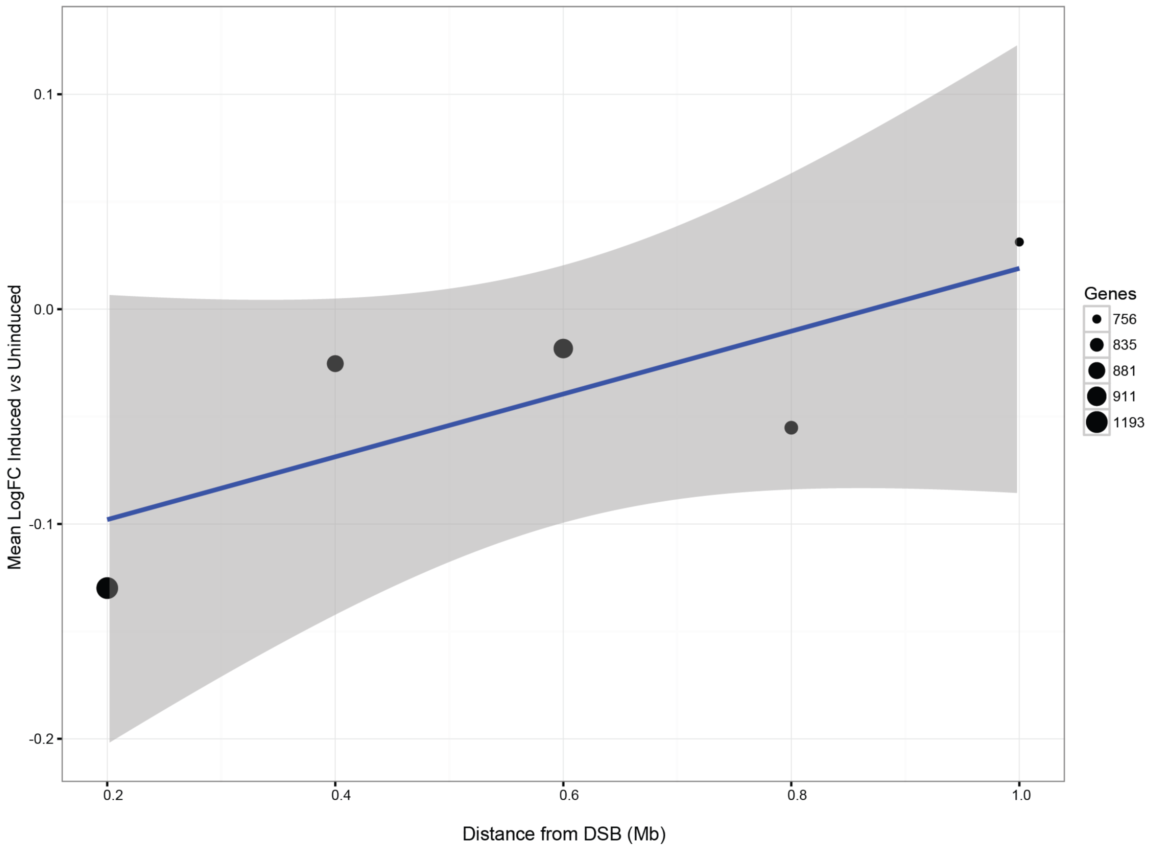

Supplementary Figure 7. Plot showing the mean log fold-change of expression in induced *vs* uninduced samples upon increasing genomic distance. Mean logarithm of the fold change was measured for each set of genes located at a certain distance from the break and normalized by the total number of sites checked. Distance from the DSB is reported in Mb on the x-axis. The diameter of each circle represents the number of genes considered (as reported in figure legend). Blue line represents linear fit to the data and grey-shaded area the standard error.

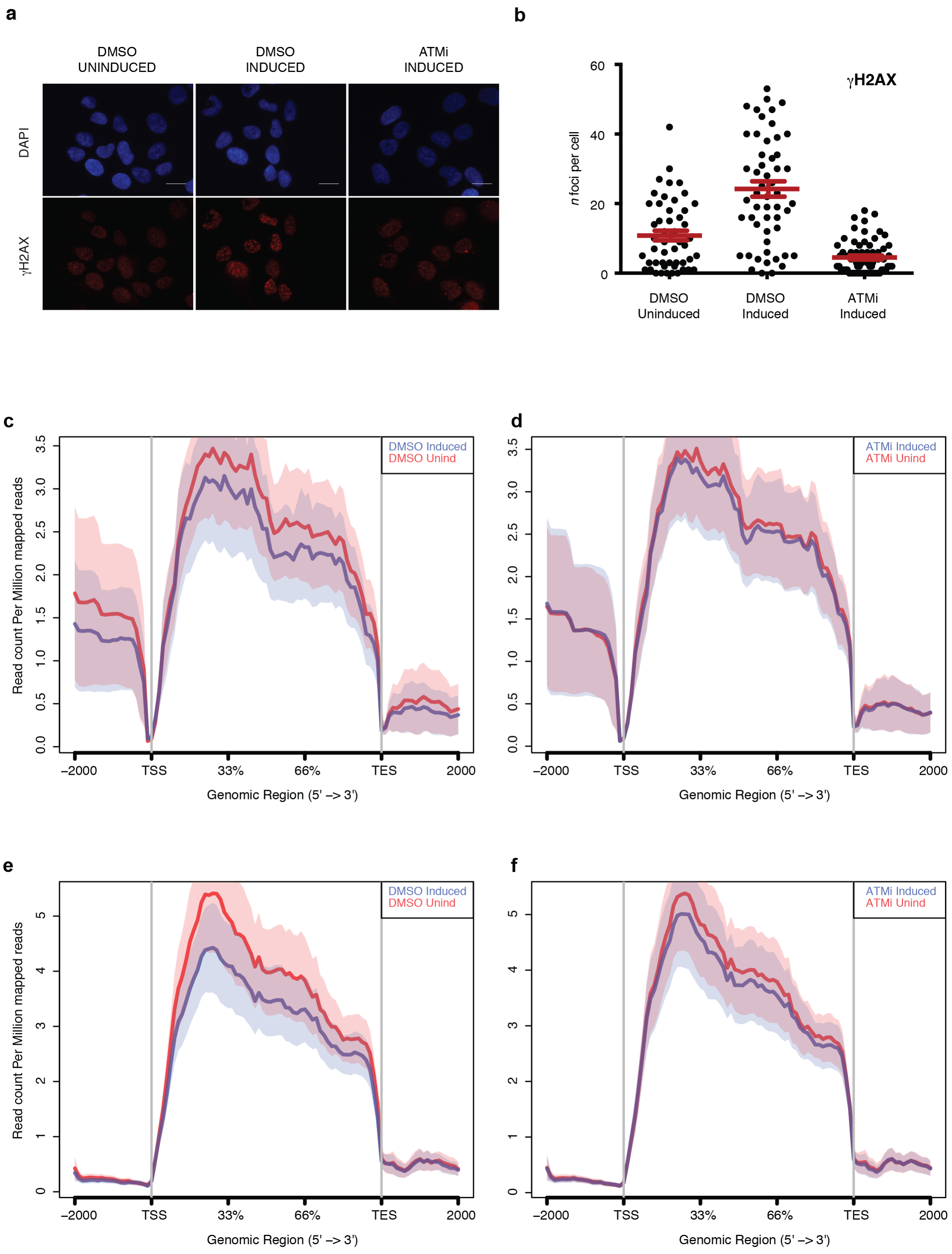

Supplementary Figure 8. (**a,b**) Representative images (**a**) and quantification analyses (**b**) of immunofluorescence for γH2AX following 4OHT treatment (induced) or mock treatment

(uninduced) in control DMSO- and ATMi-treated DIvA cells. Cells where either induced with 4OHT for 4 hours or uninduced (mock treated) and ATMi or DMSO treated; DNA was stained by DAPI. Scale bars: 20µm. DNA damage is induced by the translocation of *Asi*SI in the nucleus. (**c,d**) Coverage profile plot representing the RPM in induced (blue) and uninduced (red) samples of transcripts having cut *Asi*SI sites located upstream of their gene body in DMSO (**c**) or ATMi (**d**) treated cells: TSS = transcription start site, TES = transcription end site. Bold lines represent mean value, the semi-transparent shades around the mean curve represent the SEM. (**e,f**) Coverage profile plot representing the RPM in induced (blue) and uninduced (red) samples of transcripts having cut *Asi*SI sites located within their gene body in DMSO (**e**) or ATMi (**f**) treated cells: TSS = transcription start site, TES = transcription end site. Bold lines represent mean value, while the semi-transparent shades around the mean curve represent the SEM.

***Asi*SI sites**	***In silico***	**BLISS**	**Gold set**
**Total sites**	1219	214	74
**Sites next to genes**	780	178	65
**Genes next to sites**	780	196	75

Supplementary Table 1. Statistics for *Asi*SI sites according to *in silico* prediction, detected as cut by BLISS or detected as cut by both γH2AX and BLISS (Gold set). Sites next to genes = *Asi*SI sites that were within or adjacent (± 2Kb) to gene bodies. Genes next to sites = genes overlapping or located in proximity (± 2 Kb) to cut *Asi*SI sites.

**Control type**	**Coordinates (hg19)**	**Reference**	**BLISS**
Positive	chr9:130693170	2	YES
Positive	chr17:5973962	2	YES
Positive	chr17:80250840	2	YES
Positive	chr18:7566712	2	YES
Positive	chr1:89458596	1	YES
Positive	chr19:30019487	2	YES
Positive	chr20:30946312	2	YES
Positive	chr20:42087117	2	YES
Positive	chr21:22370445	1	YES
Positive	chr1:110036699	6	YES
Positive	chr21:33245518	2	YES
Positive	chr22:20850307	1	YES
Positive	chr22:38864101	2	YES
Positive	chr6:135819347	1	YES
Positive	chr6:90348186	2	YES
Negative	chr1:3103043	1	NO
Negative	chr6:101398544	1	NO
Negative	chr6:40555833	1	NO

Supplementary Table 2. BLISS validation based on *Asi*SI sites validated in literature. List of *Asi*SI sites validated in literature to be cut (used as Positive controls) or not cut (used as Negative controls). The efficiency of BLISS method to detect a site as cut or not cut is reported for each *Asi*SI site.

Description	Sequence 5'-3'
Forward linker for *in situ* ligation in BLISS	GCGTGATGNNNNNNNNGATCGTCGGACTGTAGAACTCTGAACCCCTATAGTGA GTCGTATTACCGGCCTCAATCGAA
Reverse linker for *in situ* ligation in BLISS	CGATTGAGGCCGGTAATACGACTCACTATAGGGGTTCAGAGTTCTACAGTCCGAC GATCNNNNNNNNCATCACGC

Supplementary Table 3. Linkers for *in situ* ligation in BLISS. Sequences of the linkers used for BLISS experiments. BLISS linkers have been annealed to form a dsDNA oligo that can be ligated to free DNA ends.

**Gene**	**Primer Orientation**	**Sequence**
MIS12	FW	GAGAGAAGATGAGGCGGTAGA
MIS12	REV	GCCAATGTCCTCAATTTGCT
GNE	FW	TTGTTACACTTATCACAGGCACAG
GNE	REV	GAGCTTCCGTGGATCAATTC
RBMXL1	FW	TCAGGACTAGTTCGCAGCAG
RBMXL1	REV	TCGAGGTGGACCTCCATAAC
KLF6	FW	AAAGCTCCCACTTGAAAGCA
KLF6	REV	CCTTCCCATGAGCATCTGTAA
KLF7	FW	GGTGCAAAGCCCTTCAAAT
KLF7	REV	CTTCATGGAGGGCAAGAT
TRIM37	FW	CAGCTGCCCCTGAAGAAG
TRIM37	REV	CGCCCACACTGGTATGCT
HUNK	FW	CCTACGCTGCACCTGAACT
HUNK	REV	CATGGCATACATGTTCACACCT
CYB561D1	FW	TTTGGGCTTCACCATCTTTC
CYB561D1	REV	CCATCAGGGGACCTGTTTT
RPLP0	FW	TTCATTGTGGGAGCAGAC
RPLP0	REV	CAGCAGTTTCTCCAGAGC
B2M	FW	TTCTGGCCTGGAGGCTATC
B2M	REV	TCAGGAAATTTGACTTTCCATTC
GFP	FW	GAGCAAGGGCGAGGAGCTGT
GFP	RV	ACGCTGAACTTGTGGCCGTTT
c-Myc	FW	TTATATTCCGGGGGTCTGC
c-Myc	RV	GGAGCTGAGTGAGGCGAGT

Supplementary Table 4. Primer sequences (5’-3’ orientation) for real-time quantitative PCR. Sequences of the primers used for qPCR experiments for tested genes and housekeeper genes used for normalization (*RPLP0* and *B2M*).

Supplementary References

1. Iacovoni, J.S. *et al.* High-resolution profiling of gammaH2AX around DNA double strand breaks in the mammalian genome. *EMBO J.* **29**, 1446-57. doi: 10.1038/emboj.2010.38. Epub 2010 Apr 1. (2010).
2. Aymard, F. *et al.* Transcriptionally active chromatin recruits homologous recombination at DNA double-strand breaks. *Nat Struct Mol Biol.* **21**, 366-74. doi: 10.1038/nsmb.2796. Epub 2014 Mar 23. (2014).
3. Lensing, S.V. *et al.* DSBCapture: in situ capture and sequencing of DNA breaks. *Nat Methods.* **13**, 855-7. doi: 10.1038/nmeth.3960. Epub 2016 Aug 15. (2016).
4. Khurana, S. *et al.* A macrohistone variant links dynamic chromatin compaction to BRCA1-dependent genome maintenance. *Cell Rep.* **8**, 1049-62. doi: 10.1016/j.celrep.2014.07.024. Epub 2014 Aug 14. (2014).
5. Soutoglou, E. *et al.* Positional stability of single double-strand breaks in mammalian cells. *Nat Cell Biol.* **9**, 675-82. Epub 2007 May 7. (2007).
6. Zhou, Y., Caron, P., Legube, G. & Paull, T.T. Quantitation of DNA double-strand break resection intermediates in human cells. *Nucleic Acids Res.* **42**, e19. doi: 10.1093/nar/gkt1309. Epub 2013 Dec 19. (2014).
